# Treatment of Middle East respiratory syndrome with a combination of lopinavir/ritonavir and interferon-β1b (MIRACLE trial): statistical analysis plan for a recursive two-stage group sequential randomized controlled trial

**DOI:** 10.1186/s13063-019-3846-x

**Published:** 2020-01-03

**Authors:** Yaseen M. Arabi, Ayed Y. Asiri, Abdullah M. Assiri, Hani A. Aziz Jokhdar, Adel Alothman, Hanan H. Balkhy, Sameera AlJohani, Shmeylan Al Harbi, Suleiman Kojan, Majed Al Jeraisy, Ahmad M. Deeb, Ziad A. Memish, Sameeh Ghazal, Sarah Al Faraj, Fahad Al-Hameed, Asim AlSaedi, Yasser Mandourah, Ghaleb A. Al Mekhlafi, Nisreen Murad Sherbeeni, Fatehi Elnour Elzein, Abdullah Almotairi, Ali Al Bshabshe, Ayman Kharaba, Jesna Jose, Abdulrahman Al Harthy, Mohammed Al Sulaiman, Ahmed Mady, Robert A. Fowler, Frederick G. Hayden, Abdulaziz Al-Dawood, Mohamed Abdelzaher, Wail Bajhmom, Mohamed A. Hussein, and the Saudi Critical Care Trials group

**Affiliations:** 1 College of Medicine, King Saud Bin Abdulaziz University for Health Sciences, King Abdullah International Medical Research Center, Riyadh, Saudi Arabia; 2 Intensive Care Department, Ministry of the National Guard - Health Affairs, ICU 1425, P.O. Box 22490, Riyadh, 11426 Saudi Arabia; 3 Prince Mohammed bin Abdulaziz Hospital, Riyadh, Saudi Arabia; 4 Infection Prevention and Control, Assistant Deputy Minister, Preventive Health, Ministry of Health, Riyadh, Saudi Arabia; 5 Deputy Minister for Public Health, Ministry of Health, Riyadh, Saudi Arabia; 6 Department of Medicine, Ministry of the National Guard - Health Affairs, Riyadh, Saudi Arabia; 7 Department of Infection Prevention and Control, Ministry of the National Guard - Health Affairs, Riyadh, Saudi Arabia; 8 Department of Pathology and Laboratory Medicine, Ministry of the National Guard - Health Affairs, Riyadh, Saudi Arabia; 9 College of Pharmacy, King Saud Bin Abdulaziz University for Health Sciences, King Abdullah International Medical Research Center, Riyadh, Saudi Arabia; 10 Pharmaceutical Care Department, Ministry of the National Guard - Health Affairs, Riyadh, Saudi Arabia; 11 King Saud Bin Abdulaziz University for Health Sciences, King Abdullah International Medical Research Center, Research Office, Riyadh, Saudi Arabia; 12 Ministry of the National Guard - Health Affairs, Riyadh, Saudi Arabia; 13 Prince Mohammed bin Abdulaziz Hospital, Ministry of Health & College of Medicine, Alfaisal University, Riyadh, Saudi Arabia; 14 Hubert Department of Global Health, Rollins School of Public Health, Emory University, Atlanta, Georgia USA; 15 College of Medicine, King Saud Bin Abdulaziz University for Health Sciences, King Abdullah International Medical Research Center, Jeddah, Saudi Arabia; 16 Intensive Care Department, Ministry of the National Guard - Health Affairs, Jeddah, Saudi Arabia; 17 Department of Infection Prevention and Control, Ministry of the National Guard - Health Affairs, Jeddah, Saudi Arabia; 18 Military Medical Services, Ministry of Defense, Prince Sultan Military Medical City, Riyadh, Saudi Arabia; 19 Department of Intensive Care Services, Prince Sultan Military Medical City, Riyadh, Saudi Arabia; 20 Infectious Diseases Division, Prince Sultan Military Medical City, Riyadh, Saudi Arabia; 21 Department of Critical Care Medicine, King Fahad Medical City, Riyadh, Saudi Arabia; 22 Department of Critical Care Medicine, King Khalid University, Aseer Central Hospital, Abha, Saudi Arabia; 23 Department of Critical Care, King Fahad Hospital, Ohoud Hospital, Al-Madinah Al-Monawarah, Saudi Arabia; 24 Department Biostatistics and Bioinformatics, King Saud Bin Abdulaziz University for Health Sciences, King Abdullah International Medical Research Center, Riyadh, Saudi Arabia; 25 Intensive Care Unit, King Saud Medical City, Riyadh, Saudi Arabia; 26 Infectious Disease, King Saud Medical City, Riyadh, Saudi Arabia; 27 Intensive Care Department, King Saud Medical City, Riyadh, Saudi Arabia; 28 Department of Anesthesiology and Intensive Care, Tanta University Hospitals, Tanta, Egypt; 29 Institute of Health Policy Management and Evaluation, University of Toronto, Toronto, Canada; 30 Department of Critical Care Medicine and Department of Medicine, Sunnybrook Hospital, Bayview Avenue, Room D478, Toronto, 2075 Canada; 31 International Severe Acute Respiratory and Emerging Infection Consortium (ISARIC), Division of Infectious Diseases and International Health, Department of Medicine, University of Virginia School of Medicine, Charlottesville, Virginia USA; 32 Critical Care Medicine Department, King Abdullah Medical Complex, Jeddah, Saudi Arabia; 33 Critical Care Medicine Department, Cairo University Hospital, Cairo, Egypt; 34 Internal Medicine Department, King Fahad General Hospital, Ministry of Health, Jeddah, Saudi Arabia

**Keywords:** Coronavirus, MERS, Antiviral, Clinical trial, Lopinavir/ritonavir, Interferon-β1b, Statistical analysis plan, Protocol

## Abstract

**Abstract:**

The MIRACLE trial (MERS-CoV Infection tReated with A Combination of Lopinavir/ritonavir and intErferon-β1b) investigates the efficacy of a combination therapy of lopinavir/ritonavir and recombinant interferon-β1b provided with standard supportive care, compared to placebo provided with standard supportive care, in hospitalized patients with laboratory-confirmed MERS. The MIRACLE trial is designed as a recursive, two-stage, group sequential, multicenter, placebo-controlled, double-blind randomized controlled trial. The aim of this article is to describe the statistical analysis plan for the MIRACLE trial. The primary outcome is 90-day mortality. The primary analysis will follow the intention-to-treat principle. The MIRACLE trial is the first randomized controlled trial for MERS treatment.

**Trial registration:**

ClinicalTrials.gov, NCT02845843. Registered on 27 July 2016.

## Background

Middle East respiratory syndrome (MERS) is a viral respiratory disease caused by the Middle East respiratory syndrome coronavirus (MERS-CoV). MERS cases continue to occur and are often associated with respiratory and multiorgan failure [1]. There is no antiviral treatment with proven efficacy at present [1, 2].

The MIRACLE trial (*M*ERS-CoV *I*nfection t*R*eated with *A C*ombination of *L*opinavir/ritonavir and int*E*rferon-β1b) is the first randomized controlled trial for MERS treatment. The study protocol has been previously published [3].

There are several challenges in a trial for treatment of a disease like MERS: (1) there is not enough information on the effect size of the lopinavir/ritonavir and interferon-β1b provided with standard supportive care compared to placebo provided with standard supportive care to conduct adequate planning for the study sample size; (2) MERS is a sporadic, unpredictable, and rare disease, which makes it difficult to plan a separate pilot study to collect the necessary information needed for the planning of the main trial. To overcome these challenges, we designed the MIRACLE trial as a recursive two-stage adaptive trial, which is a relatively new method for group sequential trials [4–7]. The approach is based on the conditional error principle, which allows for flexible and continuous adjustment of the trial parameters using data observed during prior stages without inflation of the type I error [8]. Another advantage of this method is the flexibility in setting the timing and the number of needed interim analyses. Such flexibility is necessary in a situation where recruitment rate is unpredictable and a sudden flux in recruitment of patients could happen at any time. Finally, the design takes advantage of the accumulated information throughout the trial from every single recruited patient as opposed to a traditional two-study approach (pilot followed by the main trial).

In this article, we describe the MIRACLE trial statistical analysis plan (SAP) in advance of trial completion. We identify the procedures to be followed for the primary and secondary analyses for the trial. The SAP was written by the study steering committee members led by the principal investigator, who remains blinded to both group allocation and to study results until after completing patient recruitment, patient follow-up, and completion and locking of the database. The final study report will follow the guidelines of the Consolidated Standards of Reporting Trials (CONSORT) for reporting randomized controlled trials [9, 10].

The trial is being conducted according to the standard requirements of Good Clinical Practice E6 [11]. The SAP was developed in accordance with the International Council for Harmonisation guidelines (E9 Statistical principles for clinical trials and E3 clinical study reports guidelines) [12, 13] and with the Guidelines for the Content of Statistical Analysis Plans in Clinical Trials [14].

## Methods

### Study design

The MIRACLE trial is a recursive, two-stage, group sequential, multicenter, randomized, placebo-controlled, double-blind trial. The trial includes hospitalized patients who are 18 years old or older with laboratory-confirmed MERS in addition to evidence of acute organ dysfunction that is judged related to MERS. Inclusion and exclusion criteria have been detailed in a previously published protocol manuscript [3]. Patients are randomized to receive lopinavir/ritonavir and recombinant interferon-β1b or placebo. Randomization is stratified according to center and according to whether the patients require mechanical ventilation (invasive or non-invasive) at the time of enrollment, as mechanical ventilation is a major, but pragmatic, surrogate for severity of illness. The study interventions continue for 14 days or until hospital discharge. Patients are followed up daily until day 28 or hospital discharge and then at day 90.

### Study population

**Fig. 1:**
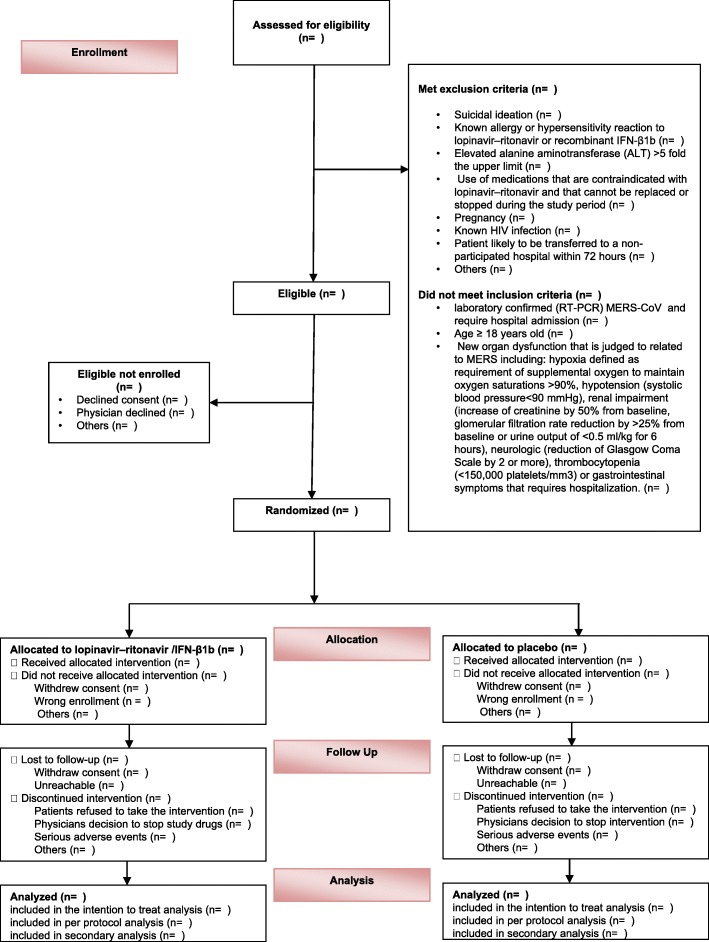
CONSORT flow chart for the MIRACLE trial

A CONSORT flow diagram of the trial progress will be constructed (Fig. 1). The number of randomized patients to each group will be reported as well as the number of randomized patients who received the interventions. We will also report the number of screened patients (defined as all hospitalized patients with MERS) who met the eligibility criteria but were not enrolled and the reasons for non-enrollment.

The *intention-to-treat population* consists of all enrolled patients whether or not they received the allocated intervention, and will be used for the primary analysis. A per-protocol analysis will be conducted for patients who received the allocated interventions (defined by any dose of the study intervention).

### Data

Baseline characteristics

Baseline characteristics will be presented for the two study groups (Additional file 1: Table S1) including age, sex, and body mass index, the presence of co-infections, nosocomial versus community-acquired MERS infection, Acute Physiology and Chronic Health Evaluation (APACHE) II scores, Sequential Organ Failure Assessment scores, and the Karnofsky Performance Status Scale score [3]. We will report comorbidities and the interventions received before randomization for the patients in each group. We will report baseline laboratory values (international normalization ratio, platelet count, hemoglobin, white blood cell count, lymphocyte count, liver enzymes, glucose, serum amylase, blood urea nitrogen, creatinine, creatine kinase, lactate) and respiratory and vital parameters in addition to the location of the patient at time of randomization.

### Intervention data

For each group we will report the time of hospital admission to randomization and the time of randomization to the first dose received of the study drugs. We will report the received study intervention and its duration for each group, in addition to the missing or incomplete doses and protocol violations (Additional file 1: Table S2 and Table S8).

### Co-interventions

We will compare any use of vasopressors, renal replacement therapy, neuromuscular blockade, mechanical ventilation, extracorporeal membrane oxygenation (ECMO), nitric oxide, prone ventilation, and tracheostomy. We will also compare the use of intravenous immunoglobulin, antiviral therapy, antibiotics, corticosteroids, and statins (Additional file 1: Table S2).

### Primary and secondary outcomes

The *primary outcome* is 90-day mortality (Additional file 1: Table S3). The primary outcome is defined as all-cause mortality after enrollment in the trial within 90 days, as either an inpatient or outpatient.

*Secondary outcomes and subgroups* are defined as presented in Table 1 and Additional file 1: Table S4, and S5).

**Table 1:** Secondary Outcomes in the MIRACLE trial

Outcome	Definition
28-day mortality	Death from any cause within 28 days of enrollment
Hospital mortality	Death from any cause in the index hospitalization
ICU mortality	Death from any cause in index ICU admission.
Sequential Organ Failure Assessment scores	SOFA score on study days 0, 3, 7, 14, 21 and 28
Supplemental oxygen-free days	Number of days within the first 28 days after enrollment when patients do not receive of supplemental oxygen. Patients who die within 28 days will be assigned the value “0”
Renal replacement therapy-free days	Number of days within the first 28 days after enrollment when patients do not receive of renal replacement therapy. Patients who die within 28 days will be assigned the value “0”
Vasopressor-free days	Number of days within the first 28 days after enrollment when patients do not receive of vasopressors. Patients who die within 28 days will be assigned the value “0”
Invasive or non-invasive mechanical ventilation-free days	Number of days within the first 28 days after enrollment when patients do not receive of mechanical ventilation. Patients who die within 28 days will be assigned the value “0”
Organ support-free days	Number of days within the first 28 days after enrollment when patients do not receive of invasive mechanical ventilation, renal replacement therapy and vasopressor. Patients who die within 28 days will be assigned the value “0”
Extracorporeal circulation support-free days	Number of days within the first 28 days in which patients are not receiving extracorporeal circulation support. Patients who die within 28 days will be assigned the value “0”
ICU-free days	Number of days in which patients are not being cared for in the ICU during the first 28 days after enrollment. Patients who die within 28 days will be assigned the value “0”
Post-randomization hospital length of stay	Number of days between randomization and discharge from the hospital. Because of the competing risk effect of death on length of stay, length of stay will be also reported for survivors alone
Renal replacement therapy at day 90	Number and percentage of patients on renal replacement therapy at day 90
Oxygen supply at day 90	Number and percentage of patients on oxygen supply at day 90
Non-invasive mechanical ventilation at day 90	Number and percentage of patients on non-invasive mechanical ventilation at day 90
Invasive mechanical ventilation at day 90	Number and percentage of patients on Invasive mechanical ventilation at day 90
Secondary laboratory outcomes	
Viral replication kinetics	upE and ORF1 cycle thresholds of blood and respiratory samples
Time to clearance from the lower respiratory tract	Number of days from randomization to MERS-CoV RNA clearance of respiratory samples defined as two negative RT-PCR results not followed by a positive one. Patients who die before clearance will be censored at the time of death
Safety outcomes	
Serious adverse event reports (SAE)	The number and percentage of reported serious adverse events any time during the study period. These SAEs include: acute pancreatitis, severe elevation of Alanine aminotransferase (ALT) to more than five-fold the upper normal limit, anaphylaxis, bleeding diathesis and others
Adverse Events	The number and percentage of adverse events graded using the Common Terminology Criteria for Adverse Events, at any time within 28 days after enrollment. The adverse drug reactions include: allergic reactions, gastrointestinal, general nervous system and others. See also Table S6
Functional outcomes	
Karnofsky score	Karnofsky Performance Status Scale for functional impairment, which is a scale from 100 (indicating “Normal,” no complaints; no evidence of disease) to 0 (indicating death) at day 90

In addition, we will compare the physiological parameters among patients treated in the treatment group and the control group.

### Statistical analysis

All analyses will be performed using SAS 9.4 with specially written code for the analysis of the primary outcome that accounts for the recursive design, as described in Chang [4].

#### Data and Safety Monitoring Board and interim analyses

A detailed interim analysis plan is reported in the MIRACLE protocol [3]. The trial is designed as a recursive, two-stage, group sequential randomized trial. The first interim analysis will be conducted when 34 subjects (17 per group) have completed 90 days of follow-up. This is about 17.5% of the total sample size needed for the classical design (a classic two-group design requires a total of 194 subjects (97 subjects per group) to have an 80% power at a significance level of 5% using a one-sided *Z* test for difference in proportion to detect 20% absolute risk reduction in 90 days mortality among subjects receiving treatment (20%) compared to a control group (40%)). A Data and Safety Monitoring Board (DSMB) will be convened to review the unblinded data (efficacy and safety) and advise on continuation or termination of the trial. The determination of the stopping boundaries in the first two-stage design was calculated using the conditional power method based on the summing stage-wise *p* values. At the first interim analysis, the DSMB will determine whether the trial should be terminated for futility or not using the following boundaries and their corresponding decisions (Table 2).

**Table 2:** Stopping boundaries in the MIRACLE trial

Boundary	Value	Decision
Efficacy sopping boundary (α_1_)	0	No stopping for efficacy
Futility stopping boundary (β_1_)	0.2	Stop the trial for futility if less than stage-wise *P*-value
Efficacy stopping boundary (α_2_)	0.2250	Stop trial for efficacy at the second stage or recalculate based on conditional power at first interim analysis

α1 is the maximum probability threshold under which the trial will be terminated early for efficacy. β1 is the maximum probability threshold above which the trial will be terminated for futility. α2 is the maximum probability threshold (the sum of the stage-wise *p*-values), above which the study will be declared as met its endpoint

#### Demographics and clinical characteristics

We will summarize and report the demographics and baseline clinical characteristics using descriptive statistics. As appropriate, the chi-square test or Fisher’s exact test will be used to compare the categorical variables, which will be reported as numbers and percentages. Student’s *t* test or the Mann–Whitney *U* test will be used as appropriate to compare the continuous variables, which will be reported as means and standard deviations or as medians and interquartile ranges.

#### Analysis of adverse events

All adverse events will be grouped using Common Terminology Criteria for Adverse Events (CTCAE) Version 4 of the National Institutes of Health (NIH) (Additional file 1: Table S6). Adverse events will be grouped into aggregate groups and reported for the entire study period (Additional file 1: Table S7). All results will be summarized in terms of frequency and percentage and will be compared across study arms using Fisher’s exact test. All results will be declared statistically significant with a *p* value < 0.05.

#### Analysis of the primary outcome and continuous planning of the trial

Let *K* be the number of stages of the current clinical trial needed to complete the trial and *i* ∈ {1, 2} be the index for the two-stage design in the *k*^*th*^ stage. Let *r*_1*ki*_ *and r*_2*ki*_ be the proportions of 90 days mortality in the standard of care and treatment group respectively. Then the *Z* test statistic for the difference in proportion can be calculated as follows:

$$ {Z}_{ki}=\frac{\delta_{ki}}{\sigma_{ki}}\sqrt{\frac{n_{ki}}{2}}, $$

$$ {\sigma}_{ki}=\sqrt{\left[{r}_{1 ki}\left(1-{r}_{1 ki}\right)+{r}_2\left(1-{r}_{2 ki}\right)\right]/2}, $$

$$ {\delta}_{ki}={r}_{1 ki-}{r}_{2 ki}, $$

where *n*_*ki*_ is the sample size per group for the *i*^*th*^ two-stage design of the *k*^*th*^ stage. In the interim analysis (i.e., at each *i* = 1 of the *k*^*th*^ two-stage), the primary outcome will be evaluated, and the trial sample size will be re-estimated for the subsequent stage based on the observed effect size using the following formula assuming a conditional power of 80% (*Pc* = 0.8) to decide if the trial should continue:

$$ {n}_{k,2}={\left[\frac{\sqrt{2}{\sigma}_{k1}}{\delta {\delta}_{k1}}\left({\Phi}^{-1}\left(1-{\alpha}_{k,2}+{p}_{k,1}\right)-{\Phi}^{-1}\left(1- Pc\right)\right)\right]}^2. $$

Here *α*_*k*, 2_ is the precalculated rejection boundary for efficacy at the second stage of the two-stage design at the *k*^*th*^ stage, and *p*_*k*, 1_ is the raw table probability corresponding to the *Z*_*ki*_ statistic. At the first interim analysis, should the data suggest that another stage of the two-stage steps is required, we will recalculate the conditional error and new boundaries will be calculated for *K* = 2. Let *β*_*k* + 1, 1_, *α*_*k* + 1, 1_ be the rejection boundaries for futility and efficacy for the first (*i* = 1) of the two-stage step of the *k*^*th*^ + 1 stage. Then the conditional error is:

$$ A\left({p}_{k,1}\right)={\alpha}_{k+1,1}+{\alpha}_{k+1,2\kern0.5em }\left({\beta}_{k+1,1}-{\alpha}_{k+1,1}\ \right)-\frac{1}{2}\left({\beta}_{k+1,1}^2-{\alpha}_{k+1,1}^2\right),k=0,1,\dots K, $$

where *A*(*p*_0, 1_) is the type I error, which is set to 0.05. The new *α*_*k* + 1, 2_ boundary for the *k*^*t*h^ + 1 stage for pre chosen *β*_*k* + 1, 1_, *α*_*k* + 1, 1_ will be calculated as follows:

$$ {\alpha}_{k+1,2}=\frac{A\left({p}_{k,1}\right)+\frac{1}{2}\left({\beta}_{k+1,1}^2-{\alpha}_{k+1,1}^2\right)-{\alpha}_{k+1,1}}{\beta_{k+1,1}-{\alpha}_{k+1,1}\ }. $$

At the end of the trial, the treatment will be declared efficacious if the calculated stage-wise ordered *p* value *p*_*k*, 2_ is less than *α*_*k*, 2_. The adjusted *p* value will be obtained using backward recursion as follows:

$$ \left\{\begin{array}{c}{P}_{K_0-1,2}=\left\{\begin{array}{c}t\kern23.5em for\ k=1,\\ {}\ {\alpha}_{k_0,1}+t\left({\beta}_{k_0,1}-{\alpha}_{k_0,1}\right)-\frac{1}{2}\left({\beta^2}_{k_0,1}-{\alpha^2}_{k_0,1}\right)\kern1.5em for\ k=2,\end{array}\right.\\ {}{P}_{i-1,2}={\alpha}_{i,1}+\left({p}_{i,1}+{p}_{i,2}\right)\left({\beta}_{i,1}-{\alpha}_{i,1}\right)-\frac{1}{2}\left({\beta^2}_{i,1}-{\alpha^2}_{i,1}\right)\kern2.75em for\ i=1,\dots, {K}_{0-1}\end{array}\right.\operatorname{} $$

where *K*_0_ is the total number of two-stage stages, and *t* is the sum of stage-wise raw *p* values. Finally, the adjusted overall 95% one-sided confidence interval will be calculated by:

$$ {\delta}_c=\underset{1\le \mathrm{i}\le {K}_0-1}{\max}\left\{{\delta}_{i,1},{\delta}_{k_0,2}\right\}, $$

where *δ*_*i*, 1_ and ${\delta}_{k_0,2}$ are the stage-wise and the last stage of the *k*th two-stage design confidence interval bound. The last stage confidence bound ${\delta}_{k_0,2}$ can be found by solving the following equation numerically for ${\delta}_{k_0,2}$:

$$ \Phi \left(\frac{\delta_{k_0,2}}{\overset{\sim }{\sigma }}\sqrt{\frac{n_{k0,1}}{2}}-{z}_{1-{p}_{k0,1}}\right)+\Phi \left(\frac{\delta_{k_0,2}}{\overset{\sim }{\sigma }}\sqrt{\frac{n_{k0,2}}{2}}-{z}_{1-{p}_{k0,2}}\right)={\alpha}_{k_0,2}, $$

where *n*_*k*0, 1_ and *n*_*k*0, 2_ are the sample sizes for the first and second stage of the last *k*^*th*^ two-stage design, and *p*_*k*0, 1_, *p*_*k*0, 2_ are the stage-wise adjusted *p* values.

In order to stay consistent with the method that was used in calculating the boundaries for the trial, we will not account for stratification in the primary outcome analysis. In general, this approach is acceptable and it preserves both type I and type II errors as long as the weighted average of the effect size stays close to the hypothesized effect size [15]. Furthermore, as long as the sample size re-estimation at the interim analysis was based on the weighted average of the effect size, the overall power of the trial will be preserved.

#### Secondary analyses of the primary outcome, secondary outcomes, and subgroups

With the exception of the analysis of the primary outcome, all other analyses will be tested using regular statistical methods and will be two-sided. A secondary adjusted analysis will be conducted using multiple logistic regression analysis, in which death within 90 days will be modeled as the dependent variable, and a set of baseline variables that are strongly believed to affect the outcome of MERS will be included as independent variables. Those variables will include at minimum the following: age, community-acquired versus hospital-acquired infection, mechanical ventilation, center, and Sequential Organ Failure Assessment score. Ninety-day median survival time will be summarized and reported using Kaplan–Meier curves and will be compared between the study groups using the log-rank test (Additional file 1: Figure S1). Analysis of secondary outcomes will be compared in the intention-to-treat cohort only. Subgroup analyses will be conducted if patient numbers permit (e.g., no fewer than five patients in subgroups of interest) in a priori defined subgroups (Additional file 1: Table S5). Multivariable logistic regression will be used to report the results of tests of interactions for these subgroups.

#### Handling of missing data

All missing data will be reviewed and characterized in terms of their pattern (e.g., missing completely at random, missing at random, etc.). For missing completely at random, all analyses will be based on a list-wise deletion approach where only observations with complete values will be considered for analysis. For variables with values missing at random, multiple imputation techniques will be utilized to impute the missing values, as suggested by Rubin [16].

#### Adjustment multiplicity testing for the secondary analyses

To adjust for multiple testing, we will use the false discovery rate (FDR) as described by Benjamini and Hochberg [17]. In this procedure all hypothesis tests will be sorted in ascending order based on their calculated *p* value. All hypothesis tests below an index *K* will be rejected, where *K* is calculated as follows:

$$ K=\mathit{\max}\left\{i:p(i)\le \frac{i}{m}.q\right\}, $$

where *i* = *m*, … ,1, *m* is the total number of tested hypotheses, and *q* = 0.05.

Additional details about the SAP are available in Additional file 2.

## Discussion

The MIRACLE trial investigates the efficacy of a combination therapy of lopinavir/ritonavir and recombinant Interferon-β1b provided with standard supportive care, compared to placebo provided with standard supportive care, in hospitalized patients with laboratory-confirmed MERS.

The first patient was enrolled in November 2016. At present, 14 sites are actively screening for eligible patients. The recruitment rate in the MIRACLE trial has been slow mainly related to the decline in the number of MERS cases in Saudi Arabia. Due to the uncertainty of the efficacy level of the treatment and the recruitment rate, the trial is designed to be a recursive, two-stage, group sequential randomized trial [4].

Several methods could be utilized to build an adaptive trial. However, most of these methods would require one to specify a priori the time and type of adjustments that need to take place in the trial. For a disease such as MERS there are many factors that could limit the ability to specify a priori those elements; thus, the recursive two-stage design is a natural choice. This type of design provides enough flexibility to introduce different adjustments while learning from the observed data without inflating the type I error.

Reporting of the SAP to the MIRACLE trial in advance of trial completion will enhance evaluation of the clinical data and support confidence in the final results and the conclusion. Prior specification of the statistical methods and outcomes analysis will facilitate unbiased analyses of these important clinical data.

### Trial status

Recruitment started in November 2016 and is currently ongoing.

## Supplementary information

**Additional file 1: Table S1.** Baseline characteristics of intention-to-treat (ITT) population. **Table S2.** Summary of interventions and co-interventions. **Table S3.** Primary outcome: 90-day mortality. **Table S4.** Secondary outcomes. **Table S5.** Subgroup analyses. **Table S6.** Classification of adverse events in the MIRACLE trial (*M*ERS-CoV *I*nfection t*R*eated with *A C*ombination of *L*opinavir/ritonavir and int*E*rferon-β1b) using the NIH Common Terminology Criteria for Adverse Events (CTCAE), Version 4.0. **Table S7.** Summary of adverse events by severity. **Table S8.** Summary of protocol violations.

**Additional file 2.** Statistical analysis plan document.

## Data Availability

The datasets generated and/or analyzed during the current study are available from the corresponding author on reasonable request.

## Abbreviations

APACHE II: Acute Physiology and Chronic Health Evaluation II
CONSORT: Consolidated Standards of Reporting Trials
CTCAE: Common Terminology Criteria for Adverse Events
DSMB: Data and Safety Monitoring Board
ECMO: Extracorporeal membrane oxygenation
MERS: Middle East respiratory syndrome
MERS-CoV: Middle East respiratory syndrome coronavirus
MIRACLE trial: *M*ERS-CoV *I*nfection t*R*eated with *A C*ombination of *L*opinavir/ritonavir and int*E*rferon-β1b
NIH: National Institutes of Health
SAP: Statistical analysis plan
SFDA: Saudi Food and Drug Authority
TEAE: Treatment-emergent adverse event
