# Supplementary material for: Treatment of Middle East respiratory syndrome with a combination of lopinavir/ritonavir and interferon-β1b (MIRACLE trial): statistical analysis plan for a recursive two-stage group sequential randomized controlled trial
Source: Trials. 2020 Jan 3;21:8. doi: 10.1186/s13063-019-3846-x (PMC6942374; doi:10.1186/s13063-019-3846-x)
Supplement: Supplementary file 1 — Additional file 1: Table S1. Baseline characteristics of intention-to-treat (ITT) population. Table S2. Summary of interventions and co-interventions. Table S3. Primary outcome: 90-day mortality. Table S4. Secondary outcomes. Table S5. Subgroup analyses. Table S6. Classification of adverse events in the MIRACLE trial (MERS-CoV Infection tReated with A Combination of Lopinavir/ritonavir and intErferon-β1b) using the NIH Common Terminology Criteria for Adverse Events (CTCAE), Version 4.0. Table S7. Summary of adverse events by severity. Table S8. Summary of protocol violations. [file 13063_2019_3846_MOESM1_ESM.docx]

**Additional file 1**

**Supplemental materials to**

**Treatment of Middle East Respiratory Syndrome with a combination of lopinavir/ritonavir and interferon-β1b (MIRACLE trial): Statistical Analysis Plan for a randomized controlled trial**

**Contents**

**Collaborators**

**Supplemental Table 1. Baseline characteristics – ITT population.**

**Supplemental Table 2.** **Summary of interventions and co-interventions.**

**Supplemental Table 3. Primary Outcome: 90-day mortality.**

**Supplemental Table 4. Secondary Outcomes.**

**Supplemental Table 5. Subgroup analyses.**

**Supplemental Table 6. Classification of Adverse Events in the MIRACLE Trial (MERS-CoV Infection Treated with a Combination of Lopinavir / Ritonavir and Interferon Beta 1B) using the NIH Common Terminology Criteria for Adverse Events (CTCAE), Version 4.0.**

**Supplemental Table 7: Summary of Adverse Events by severity.**

**Supplemental Table 8: Summary of Protocol Violations.**

| **Collaborators** |  |
| --- | --- |
| **Center** | **Names** |
| **The Saudi Critical Care Trials group** |  |
| King Saud bin Abdulaziz University for Health Sciences and King Abdullah International Medical Research Center, Riyadh, Saudi Arabia | Yaseen Arabi  Adel Alothman  Hanan Balkhy  Abdulaziz Al-Dawood  Sameera AlJohani  Shmeylan Al Harbi  Suleiman Kojan  Majed Al Jeraisy  Naif Khalaf Alharbi  Ahmad M. Deeb  Badriah AlMutairi  Jesna Jose  Mohamed A Hussein  Mohammed Al Muhaidib  Sadat Musharaf  Hala Al Anizi  Reggie Dael |
| Ministry of Health, Saudi Arabia | Abdullah M Assiri  Hani A. Aziz Jokhdar  Mohammad AlMazroa  Fawaz Al-Rasheedi |
| Prince Mohammed bin Abdulaziz Hospital, Riyadh, Saudi Arabia | Ayed Asiri  Ziad A Memish  Sameeh S Ghazal  Sarah H Alfaraj  Fahad Bafaqeeh  Mohammed Alshaikh  Dhaifallah Saud Alotaibi  Mostafa Rajab  Fatima Emieraiza P. Isdung  Chloe D. Abinal  Ruchil S. Escobanez  Carlos B. landingin  Samah Badamas  Norah Abdullatif Hawsawi  Hanan Alanazi  Anwar Ali Mohammed |
| King Fahad Medical City, Riyadh, Saudi Arabia | Abdullah Al Motairi  Mushira Al Enani  Alaa Alqurashi  Fatimah Alenezi  Nada Alkhani |
| Prince Sultan Military Medical City, Riyadh, Saudi Arabia | Ghaleb A. Almekhlafi  Yasser Mandourah  Nisreen Murad Sherbeeni  Fatehi Elnour Elzein  SHATHA ANWAR ALSAMARRAI  Rima E Mahamed  Abdulrauf Ahmed Malibary  BANDER ALANAZI  Ma. Raylin Cubio  Melvin Salunga  Shatha Moayad Awad  Maha E. Aljuhani  Adnan Alghamedi  Osama Elfaki |
| King Saud Medical City, Riyadh, Saudi Arabia | Abelrahman Al-Harthy  Mohammed Al Sulaiman  Ahmed Mady  Basheer Abdelrahman Tasmyia Asaad Gultakin Bakirova  Amany Albraiky Hamad AlShahrany  Alva Alcazar |
| King Faisal Specialist Hospital & Research Center, Riyadh, Saudi Arabia | Khalid Maghrabi |
|  |  |
| King Abdulaziz Medical City, Jeddah, Saudi Arabia | Fahad Al-Hameed  Asim AlSaedi  Ohoud Al Oraabi  Jalal Al Refai  Pansy Elsamadisi  Medhat S Hendy  Sara AbuBaker Basher |
| King Abdullah Medical Complex, Jeddah, Saudi Arabia | Muhammed Abduldhaher  Wael Bajhamoum  Mohamed Azzam  Hala Ibrahim Alnazawi  Mohammad Nassar Almadani  Mohannad Saud Alnefaie |
| King Abdulaziz Hospital-Alahsa, Saudi Arabia | Jamal Chalabi  Shahinaz Bashir  Ibraheem Al-Dossary  Saleh Al Mekhloof |
|  |  |
| King Fahad Hospital, Al-Madinah Al-Monawarah, Saudi Arabia | Ayman Kharaba  Ahmad Al Jabri |
|  |  |
| Ohoud Hospital, Al-Madinah Al-Monawarah, Saudi Arabia | Ayman Kharaba  Magdy Farid  Alawi Alaidarous  Wael Alseraihi  Husam Shahada  Jinish Shimi |
| Aseer Central Hospital, Abha, Saudi Arabia | Ali Al Bshabshe  Abdelmoniem Al Bahar  Ali Alhusin  Bensi Mathew  Ahmad Mushabab |
| King Faisal Medical Complex, Taif, Saudi Arabia | Hanadi Mohamed Ahmed Ouali  Lamya Al Zubaidi  Rhea Mae Gesulga  Badr Ali Al Harthy  Abed Suryeh Algothemi  Rinu Mary Raju Philip  Rajani Rajan |
| Dammam Medical Complex, Dammam, Saudi Arabia | Shehab Suliman  Hajer Al Dossary  Mohammed Alnabi |
|  |  |
| King Fahad Hospital - Hofuf | Mahmoud Albagshi |
|  |  |
| King Khalid Hospital, Najran, Saudi Arabia | Abdulhadi Bin Eshaq |
|  |  |
| University of Toronto, Sunnybrook Health Sciences Centre, Toronto, Canada | Robert A Fowler |
| University of Virginia School of Medicine, Charlottesville, VA, USA | Frederick G Hayden |
|  |  |

**Table S1:** Baseline characteristics – ITT population.

| **Baseline characteristics** |  |  |
| --- | --- | --- |
| **Variable** | **Intervention Group (N=XXXX)** | **Placebo Group (N=XXXX)** |
| Age (Years) - mean (SD) | xx (xx.x) | xx (xx.x) |
| Male sex - n (%) | xxxx (xx.x) | xxxx (xx.x) |
| BMI (kg/m^2^) - mean (SD) | xx (xx.x) | xx (xx.x) |
|  |  |  |
| Community-acquired acquisition - n (%) | xxxx (xx.x) | xxxx (xx.x) |
| Nosocomial acquisition - n (%) | xxxx (xx.x) | xxxx (xx.x) |
| Other pathogen infections - n (%) | xxxx (xx.x) | xxxx (xx.x) |
|  |  |  |
| APACHE II - mean (SD) | xx (xx.x) | xx (xx.x) |
| APACHE III - mean (SD) | xxxx (xx.x) | xxxx (xx.x) |
| SOFA score, median (Q1 ,Q3) | xxxx (xx.x) | xxxx (xx.x) |
| Karnofsky score - median (Q1 ,Q3) | xx (xx, xx) | xx (xx, xx) |
|  |  |  |
| Intervention before randomization - n (%) |  |  |
| Renal replacement therapy | xxxx (xx.x) | xxxx (xx.x) |
| Vasopressor | xxxx (xx.x) | xxxx (xx.x) |
| Invasive mechanical ventilation | xxxx (xx.x) | xxxx (xx.x) |
| Non-invasive mechanical ventilation | xxxx (xx.x) | xxxx (xx.x) |
| Neuromuscular blockade | xxxx (xx.x) | xxxx (xx.x) |
| Extra corporeal membrane oxygenation(ECMO) | xxxx (xx.x) | xxxx (xx.x) |
| Nitric oxide | xxxx (xx.x) | xxxx (xx.x) |
| Prone ventilation | xxxx (xx.x) | xxxx (xx.x) |
| Tracheostomy | xxxx (xx.x) | xxxx (xx.x) |
| Antiviral | xxxx (xx.x) | xxxx (xx.x) |
| Antibiotics | xxxx (xx.x) | xxxx (xx.x) |
| Corticosteroids | xxxx (xx.x) | xxxx (xx.x) |
|  |  |  |
| Comorbidities - n (%) |  |  |
| Any chronic comorbidity | xxxx (xx.x) | xxxx (xx.x) |
| Chronic cardiac disease, including congenital heart disease | xxxx (xx.x) | xxxx (xx.x) |
| Chronic pulmonary disease | xxxx (xx.x) | xxxx (xx.x) |
| Chronic renal disease | xxxx (xx.x) | xxxx (xx.x) |
| Mild, moderate or severe liver disease | xxxx (xx.x) | xxxx (xx.x) |
| Chronic neurological disease, hemiplegia or paraplegia, or dementia | xxxx (xx.x) | xxxx (xx.x) |
| On immunosuppressant therapy or organ transplantation | xxxx (xx.x) | xxxx (xx.x) |
| Diabetes with chronic complications | xxxx (xx.x) | xxxx (xx.x) |
| Any malignancy including leukemia or lymphoma and metastatic solid tumor | xxxx (xx.x) | xxxx (xx.x) |
| AIDS/HIV | xxxx (xx.x) | xxxx (xx.x) |
| Rheumatologic diseases | xxxx (xx.x) | xxxx (xx.x) |
| Others | xxxx (xx.x) | xxxx (xx.x) |
|  |  |  |
| Laboratory results prior to randomization - median (Q1 ,Q3) |  |  |
| INR (highest) | xx (xx, xx) | xx (xx, xx) |
| Platelets (109/L) | xx (xx, xx) | xx (xx, xx) |
| Hemoglobin (g/L) | xx (xx, xx) | xx (xx, xx) |
| White Blood Cell count x109/L | xx (xx, xx) | xx (xx, xx) |
| Lymphocyte count x109/L | xx (xx, xx) | xx (xx, xx) |
| AST units/L | xx (xx, xx) | xx (xx, xx) |
| ALT units/L | xx (xx, xx) | xx (xx, xx) |
| GGT units/L | xx (xx, xx) | xx (xx, xx) |
| Glucose (mmol/L) | xx (xx, xx) | xx (xx, xx) |
| Bilirubin level (µmol l/L) | xx (xx, xx) | xx (xx, xx) |
| Serum amylase units/L | xx (xx, xx) | xx (xx, xx) |
| BUN (µmol /L) | xx (xx, xx) | xx (xx, xx) |
| Creatinine (µmol /L) | xx (xx, xx) | xx (xx, xx) |
| CK units/L | xx (xx, xx) | xx (xx, xx) |
| Lactate mmol/L | xx (xx, xx) | xx (xx, xx) |
| Serum cortisol (nmol/L) | xx (xx, xx) | xx (xx, xx) |
| ACTH (pg/mL) | xx (xx, xx) | xx (xx, xx) |
| TSH (mIU/L) | xx (xx, xx) | xx (xx, xx) |
| T-3 (pmol/L) | xx (xx, xx) | xx (xx, xx) |
| T-4 (pmol/L) | xx (xx, xx) | xx (xx, xx) |
|  |  |  |
| Respiratory parameters, median (Q1, Q3) |  |  |
| Pao2/Fio2 ratio | xx (xx, xx) | xx (xx, xx) |
| Paco2 (mmHg) | xx (xx, xx) | xx (xx, xx) |
| pH | xx (xx, xx) | xx (xx, xx) |
| Tidal volume (mL) | xx (xx, xx) | xx (xx, xx) |
| Positive end-expiratory pressure (cm H2O) | xx (xx, xx) | xx (xx, xx) |
| Number of quadrants with infiltrates on chest radiograph | xx (xx, xx) | xx (xx, xx) |
|  |  |  |
| Vital parameters, median (Q1, Q3) |  |  |
| Temperature C | xx (xx, xx) | xx (xx, xx) |
| Respiratory rate | xx (xx, xx) | xx (xx, xx) |
| Systolic BP(mmHg) | xx (xx, xx) | xx (xx, xx) |
| Highest heart rate | xx (xx, xx) | xx (xx, xx) |
| Lowest mean arterial pressure (mmHg) | xx (xx, xx) | xx (xx, xx) |
| Glasgow coma scale score | xx (xx, xx) | xx (xx, xx) |
| Urine output (mL/d) | xx (xx, xx) | xx (xx, xx) |
|  |  |  |
| Location at time of randomization - n (%) |  |  |
| ER | xxxx (xx.x) | xxxx (xx.x) |
| Ward | xxxx (xx.x) | xxxx (xx.x) |
| ICU | xxxx (xx.x) | xxxx (xx.x) |

For continuous variables, mean (SD) or median (Q1, Q3) will be summarized as appropriate.**Table S2:** Summary of interventions and co-interventions.

| **Variable** | **Intervention Group (N=XXXX)** | **Placebo Group (N=XXXX)** | **P-value** |
| --- | --- | --- | --- |
| Time of admission to randomization, (d), median (Q1, Q3) | xx (xx, xx) | xx (xx, xx) | x.xxx |
| Time of randomization to the first dose received from study drugs, (d), median (Q1, Q3) | xx (xx, xx) | xx (xx, xx) | x.xxx |
| Time of the first positive RT-PCR to the first dose received from study drugs, (d), median (Q1, Q3) | xx (xx, xx) | xx (xx, xx) | x.xxx |
| Received study intervention, n (%) | xxxx (xx.x) | xxxx (xx.x) | x.xxx |
|  |  |  |  |
| Missing/incomplete doses, n (%) |  |  |  |
| Patients refused to take the intervention | xxxx (xx.x) | xxxx (xx.x) | x.xxx |
| Physicians decision to stop the study drugs | xxxx (xx.x) | xxxx (xx.x) | x.xxx |
| Others | xxxx (xx.x) | xxxx (xx.x) | x.xxx |
|  |  |  |  |
| Number of lopinavir-ritonavir/placebo doses, n (%) | xxxx (xx.x) | xxxx (xx.x) | x.xxx |
| Lopinavir-ritonavir/placebo duration, (d), median (Q1, Q3) | xx (xx, xx) | xx (xx, xx) | x.xxx |
| Number of interferon-β1b/placebo injection doses, n (%) | xxxx (xx.x) | xxxx (xx.x) | x.xxx |
| Interferon-β1b/placebo injections duration, (d), median (Q1, Q3) | xx (xx, xx) | xx (xx, xx) | x.xxx |
|  |  |  |  |
| Protocol violation, n (%) | xxxx (xx.x) | xxxx (xx.x) | x.xxx |
|  |  |  |  |
| Vasopressors - n (%) | xxxx (xx.x) | xxxx (xx.x) | x.xxx |
| Dopamine (ug/kg/min) - mean (SD) | xxxx (xx.x) | xxxx (xx.x) | x.xxx |
| Norepinephrine (ug/kg/min) - mean (SD) | xxxx (xx.x) | xxxx (xx.x) | x.xxx |
| Epinephrine (ug/kg/min) - mean (SD) | xxxx (xx.x) | xxxx (xx.x) | x.xxx |
| Phenylephrine (ug/kg/min) - mean (SD) | xxxx (xx.x) | xxxx (xx.x) | x.xxx |
| Vasopressin (U/min) - mean (SD) | xxxx (xx.x) | xxxx (xx.x) | x.xxx |
| Dobutamine (ug/kg/min) - mean (SD) | xxxx (xx.x) | xxxx (xx.x) | x.xxx |
| Milrinone (ug/kg/min) - mean (SD) | xxxx (xx.x) | xxxx (xx.x) | x.xxx |
|  |  |  |  |
| Renal replacement therapy - n (%) | xxxx (xx.x) | xxxx (xx.x) | x.xxx |
| Neuromuscular blockade - n (%) | xxxx (xx.x) | xxxx (xx.x) | x.xxx |
| Invasive mechanical intubation - n (%) | xxxx (xx.x) | xxxx (xx.x) | x.xxx |
| Non-invasive mechanical intubation - n (%) | xxxx (xx.x) | xxxx (xx.x) | x.xxx |
| Extra corporeal membrane oxygenation (ECMO) - n (%) | xxxx (xx.x) | xxxx (xx.x) | x.xxx |
| Nitric oxide - n (%) | xxxx (xx.x) | xxxx (xx.x) | x.xxx |
| Prone ventilation - n (%) | xxxx (xx.x) | xxxx (xx.x) | x.xxx |
| Tracheostomy - n (%) | xxxx (xx.x) | xxxx (xx.x) | x.xxx |
| Intravenous immunoglobin - n (%) | xxxx (xx.x) | xxxx (xx.x) | x.xxx |
|  |  |  |  |
| Antiviral - n (%) | xxxx (xx.x) | xxxx (xx.x) | x.xxx |
| Oseltamavir - n (%) | xxxx (xx.x) | xxxx (xx.x) | x.xxx |
| Ribavirin - n (%) | xxxx (xx.x) | xxxx (xx.x) | x.xxx |
| Interferon - n (%) | xxxx (xx.x) | xxxx (xx.x) | x.xxx |
| Others - n (%) | xxxx (xx.x) | xxxx (xx.x) | x.xxx |
|  |  |  |  |
| Antibiotics - n (%) | xxxx (xx.x) | xxxx (xx.x) | x.xxx |
|  |  |  |  |
| Corticosteroids - n (%) | xxxx (xx.x) | xxxx (xx.x) | x.xxx |
| Duration (d), median (Q1, Q3) | xx (xx, xx) | xx (xx, xx) | x.xxx |
| Hydrocortisone - n (%) | xxxx (xx.x) | xxxx (xx.x) | x.xxx |
| Methylprednisolone - n (%) | xxxx (xx.x) | xxxx (xx.x) | x.xxx |
| Dexamethasone - n (%) | xxxx (xx.x) | xxxx (xx.x) | x.xxx |
| Others - n (%) | xxxx (xx.x) | xxxx (xx.x) | x.xxx |
|  |  |  |  |
| Statins - n (%) | xxxx (xx.x) | xxxx (xx.x) | x.xxx |

| **Table S3:** Primary Outcome: 90-day mortality. |  | | | |  | | |
| --- | --- | --- | --- | --- | --- | --- | --- |
| **Primary outcome** | **ITT Population** | | | | **PP Population** | | |
| **Variable** | **Intervention Group (N=XXXX)** | | **Placebo Group (N=XXXX)** | **P-value** | **Intervention Group (N=XXXX)** | **Placebo Group (N=XXXX)** | **P-value** |
| 90-day mortality, n (%) | xx/xxxx (xx.x) | | xx/xxxx (xx.x) | x.xxx | xx/xxxx (xx.x) | xx/xxxx (xx.x) | x.xxx |
| Relative risk, (95% CI) | xx.x (xx.x, xx.x) | | |  | xx.x (xx.x, xx.x) | |  |
| Days to event – Median* (Q1,Q3) | xx (xx, xx) | xx (xx, xx) | | x.xxx | xx (xx, xx) | xx (xx, xx) | x.xxx |
| Adjusted odds ratio (95% CI) | xx.x (xx.x, xx.x) | | | x.xxx | xx.x (xx.x, xx.x) | | x.xxx |
| Using survival analysis.* |  | | |  |  | |  |

**Table S4:** Secondary Outcomes.

| **Characteristic** | **Intervention Group (N=XXXX)** | **Placebo Group (N=XXXX)** | **Relative Risk, (95% CI)** | **P-value** |
| --- | --- | --- | --- | --- |
| 28-day mortality, n (%) | xxxx (xx.x) | xxxx (xx.x) | x.xx (x.xx , x.xx) | x.xxx |
| ICU mortality, n (%) | xxxx (xx.x) | xxxx (xx.x) | x.xx (x.xx , x.xx) | x.xxx |
| Hospital mortality, n (%) | xxxx (xx.x) | xxxx (xx.x) | x.xx (x.xx , x.xx) | x.xxx |
|  |  |  |  |  |
| Renal replacement therapy at day 90 - n (%) | xxxx (xx.x) | xxxx (xx.x) | x.xx (x.xx , x.xx) | x.xxx |
| Oxygen supply at day 90, n (%) | xxxx (xx.x) | xxxx (xx.x) | x.xx (x.xx , x.xx) | x.xxx |
| Non-invasive mechanical ventilation at day 90, n (%) | xxxx (xx.x) | xxxx (xx.x) | x.xx (x.xx , x.xx) | x.xxx |
| Invasive mechanical ventilation at day 90, n (%) | xxxx (xx.x) | xxxx (xx.x) | x.xx (x.xx , x.xx) | x.xxx |
| Infection episodes, n (%) | xxxx (xx.x) | xxxx (xx.x) | x.xx (x.xx , x.xx) | x.xxx |
|  |  |  |  |  |
| Sequential Organ Failure Assessment (SOFA) scores, median (Q1 ,Q3) |  |  |  |  |
| SOFA day 3 | xx (xx, xx) | xx (xx, xx) |  | x.xxx |
| SOFA day 7 | xx (xx, xx) | xx (xx, xx) |  | x.xxx |
| SOFA day 14 | xx (xx, xx) | xx (xx, xx) |  | x.xxx |
| SOFA day 21 | xx (xx, xx) | xx (xx, xx) |  | x.xxx |
| SOFA day 28 | xx (xx, xx) | xx (xx, xx) |  | x.xxx |
|  |  |  |  |  |
| Organ support outcomes, median (Q1 ,Q3) |  |  |  |  |
| Supplemental oxygen-free days | xx (xx, xx) | xx (xx, xx) |  | x.xxx |
| Renal replacement therapy-free days | xx (xx, xx) | xx (xx, xx) |  | x.xxx |
| Vasopressor-free days | xx (xx, xx) | xx (xx, xx) |  | x.xxx |
| Invasive or non-invasive mechanical ventilation free days | xx (xx, xx) | xx (xx, xx) |  | x.xxx |
| Extracorporeal circulation support-free days | xx (xx, xx) | xx (xx, xx) |  | x.xxx |
| Organ support-free days | xx (xx, xx) | xx (xx, xx) |  | x.xxx |
|  |  |  |  |  |
| ICU-free days and hospital length of stay, days, median (Q1 ,Q3) |  |  |  |  |
| ICU-free days | xx (xx, xx) | xx (xx, xx) |  | x.xxx |
| Hospital length of stay | xx (xx, xx) | xx (xx, xx) |  | x.xxx |
|  |  |  |  |  |
| Viral load |  |  |  |  |
| Ct value (respiratory), median (Q1 ,Q3) |  |  |  |  |
| upE | xx (xx, xx) | xx (xx, xx) |  | x.xxx |
| ORF1 | xx (xx, xx) | xx (xx, xx) |  | x.xxx |
| Ct value (blood), median (Q1 ,Q3) |  |  |  |  |
| upE | xx (xx, xx) | xx (xx, xx) |  | x.xxx |
| ORF1 | xx (xx, xx) | xx (xx, xx) |  | x.xxx |
| MERS-CoV RNA clearance, n (%) | xxxx (xx.x) | xxxx (xx.x) | x.xx (x.xx , x.xx) | x.xxx |
|  |  |  |  |  |
| Time to MERS-CoV RNA clearance, (d), median (Q1 ,Q3) | xx (xx, xx) | xx (xx, xx) |  | x.xxx |
| MERS-CoV RNA clearance among 90-d survivors, n (%) | xxxx (xx.x) | xxxx (xx.x) | x.xx (x.xx , x.xx) | x.xxx |
| Time to MERS-CoV RNA clearance among 90-d survivors, d, median (Q1 ,Q3) | xx (xx, xx) | xx (xx, xx) |  | x.xxx |
|  |  |  |  |  |
| Serious adverse events, n (%) |  |  |  |  |
| Acute pancreatitis | xxxx (xx.x) | xxxx (xx.x) | x.xx (x.xx , x.xx) | x.xxx |
| Severe elevation of ALT to more than five-fold the upper normal limit | xxxx (xx.x) | xxxx (xx.x) | x.xx (x.xx , x.xx) | x.xxx |
| Anaphylaxis | xxxx (xx.x) | xxxx (xx.x) | x.xx (x.xx , x.xx) | x.xxx |
| Bleeding diathesis | xxxx (xx.x) | xxxx (xx.x) | x.xx (x.xx , x.xx) | x.xxx |
| Others | xxxx (xx.x) | xxxx (xx.x) | x.xx (x.xx , x.xx) | x.xxx |
|  |  |  |  |  |
| Adverse drug reactions, n (%) |  |  |  |  |
| Allergic reactions | xxxx (xx.x) | xxxx (xx.x) | x.xx (x.xx , x.xx) | x.xxx |
| Gastrointestinal | xxxx (xx.x) | xxxx (xx.x) | x.xx (x.xx , x.xx) | x.xxx |
| General nervous system | xxxx (xx.x) | xxxx (xx.x) | x.xx (x.xx , x.xx) | x.xxx |
| Others | xxxx (xx.x) | xxxx (xx.x) | x.xx (x.xx , x.xx) | x.xxx |
|  |  |  |  |  |
| Functional outcome |  |  |  |  |
| Karnofsky score at Day 90, median (Q1, Q3) | xx (xx, xx) | xx (xx, xx) |  | x.xxx |

**Table S5:** Subgroup analyses.

|  | **90-day mortality** | | | | |
| --- | --- | --- | --- | --- | --- |
|  | **Intervention Group (N=XXXX)** | **Placebo Group (N=XXXX)** | **RR (95% CI)** | **P-value** | **P-value for interaction** |
| Mechanical ventilation at day 0 | xxxx/xxxx (xx.x) | xxxx/xxxx (xx.x) | x.xx (x.xx, x.xx) | x.xxx | x.xxx |
| No mechanical ventilation at day 0 | xxxx/xxxx (xx.x) | xxxx/xxxx (xx.x) | x.xx (x.xx, x.xx) | x.xxx |  |
|  |  |  |  |  |  |
| High APACHEII at day 0 | xxxx/xxxx (xx.x) | xxxx/xxxx (xx.x) | x.xx (x.xx, x.xx) | x.xxx | x.xxx |
| Low APACHEII at day 0 | xxxx/xxxx (xx.x) | xxxx/xxxx (xx.x) | x.xx (x.xx, x.xx) | x.xxx |  |
|  |  |  |  |  |  |
| Vasopressors at day 0 | xxxx/xxxx (xx.x) | xxxx/xxxx (xx.x) | x.xx (x.xx, x.xx) | x.xxx | x.xxx |
| No vasopressors at day 0 | xxxx/xxxx (xx.x) | xxxx/xxxx (xx.x) | x.xx (x.xx, x.xx) | x.xxx |  |
|  |  |  |  |  |  |
| Renal replacement therapy at day 0 | xxxx/xxxx (xx.x) | xxxx/xxxx (xx.x) | x.xx (x.xx, x.xx) | x.xxx | x.xxx |
| No renal replacement therapy at day 0 | xxxx/xxxx (xx.x) | xxxx/xxxx (xx.x) | x.xx (x.xx, x.xx) | x.xxx |  |
|  |  |  |  |  |  |
| ≤ 7 days between onset of symptoms to enrollment | xxxx/xxxx (xx.x) | xxxx/xxxx (xx.x) | x.xx (x.xx, x.xx) | x.xxx | x.xxx |
| > 7 days between onset of symptoms to enrollment | xxxx/xxxx (xx.x) | xxxx/xxxx (xx.x) | x.xx (x.xx, x.xx) | x.xxx |  |

**Table S6:** Classification of Adverse Events in the MIRACLE Trial (MERS-CoV Infection Treated with a Combination of Lopinavir / Ritonavir and Interferon Beta 1B) using the NIH Common Terminology Criteria for Adverse Events (CTCAE), Version 4.0.

|  | **Grade** | | | | |
| --- | --- | --- | --- | --- | --- |
| **Adverse Event** | **1** | **2** | **2** | **4** | **5** |
| Anemia | Hemoglobin (Hgb) <LLN -  10.0 g/dL; <LLN - 6.2 mmol/L; <LLN - 100 g/L | Hgb <10.0 - 8.0 g/dL; <6.2 -  4.9 mmol/L; <100 - 80g/L | Hgb <8.0 g/dL; <4.9 mmol/L;  <80 g/L; transfusion indicated | Life-threatening  consequences; urgent  intervention indicated | Death |
| Diarrhea | Increase of <4 stools per day  over baseline; mild increase in ostomy output compared to baseline | Increase of 4 - 6 stools per  day over baseline; moderate  increase in ostomy output  compared to baseline | Increase of >=7 stools per day over baseline; incontinence; hospitalization indicated; severe increase in ostomy output compared to baseline; limiting self-care ADL | Life-threatening  consequences; urgent  intervention indicated | Death |
| Allergic reaction | Transient flushing or rash,  drug fever <38 degrees C  (<100.4 degrees F);  intervention not indicated | Intervention or infusion  interruption indicated;  responds promptly to  symptomatic treatment (e.g., antihistamines, NSAIDS, narcotics); prophylactic medications indicated for <=24 hrs | Prolonged (e.g., not rapidly  responsive to symptomatic  medication and/or brief  interruption of infusion);  recurrence of symptoms  following initial improvement;  hospitalization indicated for  clinical sequelae (e.g., renal  impairment, pulmonary  infiltrates) | Life-threatening  consequences; urgent  intervention indicated | Death |
| Local skin necrosis at the injection site | Asymptomatic or mild  symptoms; clinical or  diagnostic observations only;  intervention not indicated | Moderate; minimal, local or  noninvasive intervention  indicated; limiting age appropriate  instrumental ADL | Severe or medically significant  but not immediately life-threatening;  hospitalization or  prolongation of existing  hospitalization indicated;  disabling; limiting self-care  ADL | Life-threatening consequences; urgent  intervention indicated | Death |
| Tongue Edema | Asymptomatic or mild  symptoms; clinical or  diagnostic observations only;  intervention not indicated | Moderate; minimal, local or  noninvasive intervention  indicated; limiting age appropriate  instrumental ADL | Severe or medically significant  but not immediately life-threatening;  hospitalization or  prolongation of existing  hospitalization indicated;  disabling; limiting self-care  ADL | Life-threatening consequences; urgent  intervention indicated | Death |
| Dyspnea | Shortness of breath with moderate exertion | Shortness of breath with minimal exertion; limiting instrumental ADL | Shortness of breath at rest; limiting self care ADL | Life-threatening consequences; urgent intervention indicated | Death |
| Urticaria | Urticarial lesions covering <10% BSA; topical intervention indicated | Urticarial lesions covering 10 - 30% BSA; oral intervention indicated | Urticarial lesions covering >30% BSA; IV intervention indicated | - | - |
| Alanine aminotransferase  increased | >ULN - 3.0 x ULN | >3.0 - 5.0 x ULN | >5.0 - 20.0 x ULN | >20.0 x ULN | - |
| Aspartate aminotransferase  increased | >ULN - 3.0 x ULN | >3.0 - 5.0 x ULN | >5.0 - 20.0 x ULN | >20.0 x ULN | - |
| Blood bilirubin increased | >ULN - 1.5 x ULN | >1.5 - 3.0 x ULN | >3.0 - 10.0 x ULN | >10.0 x ULN | - |
|  |  |  |  |  |  |
| CPK increased | >ULN - 2.5 x ULN | >2.5 x ULN - 5 x ULN | >5 x ULN - 10 x ULN | >10 x ULN | - |
| Creatinine increased | >1 - 1.5 x baseline; >ULN -  1.5 x ULN | >1.5 - 3.0 x baseline; >1.5 -  3.0 x ULN | >3.0 baseline; >3.0 - 6.0 x  ULN | >6.0 x ULN | - |
| Electrocardiogram QT  corrected interval prolonged | QTc 450 - 480 ms | QTc 481 - 500 ms | QTc >= 501 ms on at least  two separate ECGs | QTc >= 501 or >60 ms  change from baseline and  Torsade de pointes or  polymorphic ventricular  tachycardia or signs / symptoms of serious  arrhythmia | - |
| GGT increased | >ULN - 2.5 x ULN | >2.5 - 5.0 x ULN | >5.0 - 20.0 x ULN | >20.0 x ULN | - |
| INR increased | >1 - 1.5 x ULN; >1 - 1.5 times above baseline if on  anticoagulation | >1.5 - 2.5 x ULN; >1.5 - 2.5  times above baseline if on  anticoagulation | >2.5 x ULN; >2.5 times above baseline if on anticoagulation | - | - |
| Lymphocyte count decreased | <LLN - 800/mm3; <LLN - 0.8  x 10e9 /L | <800 - 500/mm3; <0.8 - 0.5 x 10e9 /L | <500 - 200/mm3; <0.5 - 0.2 x 10e9 /L | <200/mm3; <0.2 x 10e9 /L | - |
| Neutrophil count decreased | <LLN - 1500/mm3; <LLN - 1.5 x 10e9 /L | <1500 - 1000/mm3; <1.5 - 1.0 x 10e9 /L | <1000 - 500/mm3; <1.0 - 0.5 x 10e9 /L | <500/mm3; <0.5 x 10e9 /L | - |
| Platelet count decreased | <LLN - 75,000/mm3; <LLN -  75.0 x 10e9 /L | <75,000 - 50,000/mm3; <75.0 - 50.0 x 10e9 /L | <50,000 - 25,000/mm3; <50.0 - 25.0 x 10e9 /L | <25,000/mm3; <25.0 x 10e9/L | - |
| Serum amylase increased | >ULN - 1.5 x ULN | >1.5 - 2.0 x ULN | >2.0 - 5.0 x ULN | >5.0 x ULN | - |
| White blood cell decreased | <LLN - 3000/mm3; <LLN - 3.0 x 10e9 /L | <3000 - 2000/mm3; <3.0 - 2.0x 10e9 /L | <2000 - 1000/mm3; <2.0 - 1.0 x 10e9 /L | <1000/mm3; <1.0 x 10e9 /L | - |
| Dysgeusia | Altered taste but no change in diet | Altered taste with change in diet (e.g., oral supplements); noxious or unpleasant taste; loss of taste | - | - | - |
| Nausea | Loss of appetite without alteration in eating habits | Oral intake decreased without significant weight loss, dehydration or malnutrition | Inadequate oral caloric or fluid intake; tube feeding, TPN, or hospitalization indicated | - | - |
| Vomiting | Intervention not indicated | Outpatient IV hydration; medical intervention indicated | Tube feeding, TPN, or hospitalization indicated | Life-threatening consequences | Death |
| Abdominal Pain | Mild pain | Moderate pain; limiting instrumental ADL | Severe pain; limiting self care ADL | - | - |
| Headache | Mild pain | Moderate pain; limiting  instrumental ADL | Severe pain; limiting self-care ADL | - | - |
| Depression | Mild depressive symptoms | Moderate depressive  symptoms; limiting  instrumental ADL | Severe depressive symptoms;  limiting self-care ADL;  hospitalization not indicated | Life-threatening  consequences, threats of  harm to self or others;  hospitalization indicated | Death |
| Insomnia | Mild difficulty falling asleep,  staying asleep or waking up  early | Moderate difficulty falling  asleep, staying asleep or  waking up early | Severe difficulty in falling  asleep, staying asleep or  waking up early | - | - |
| Mania | Mild manic symptoms (e.g.,  elevated mood, rapid  thoughts, rapid speech,  decreased need for sleep) | Moderate manic symptoms  (e.g., relationship and work  difficulties; poor hygiene) | Severe manic symptoms (e.g., hypomania; major sexual or financial indiscretions); hospitalization not indicated | Life-threatening  consequences, threats of  harm to self or others;  hospitalization indicated | Death |
| Psychosis | Mild psychotic symptoms | Moderate psychotic symptoms (e.g., disorganized speech;  impaired reality testing) | Severe psychotic symptoms  (e.g., paranoid; extreme  disorganization);  hospitalization not indicated | Life-threatening  consequences, threats of  harm to self or others;  hospitalization indicated | Death |
| Bronchospasm | Mild symptoms; intervention  not indicated | Symptomatic; medical  intervention indicated; limiting  instrumental ADL | Limiting self-care ADL; oxygen  saturation decreased | Life-threatening respiratory or hemodynamic compromise;  intubation or urgent  intervention indicated | Death |
| - Other, specify | Asymptomatic or mild  symptoms; clinical or  diagnostic observations only;  intervention not indicated | Moderate; minimal, local or  noninvasive intervention  indicated; limiting age appropriate  instrumental ADL | Severe or medically significant  but not immediately life-threatening;  hospitalization or  prolongation of existing  hospitalization indicated;  disabling; limiting self-care  ADL | Life-threatening consequences; urgent  intervention indicated | Death |

**Table S7:** Summary of Adverse Events by severity.

| **Adverse Event** | **Grade** | **Intervention Group (N=XXX)**  **n (%)** | **Placebo Group (N=XXX)**  **n (%)** |
| --- | --- | --- | --- |
| Adverse Event 1 | Grade 1 | xx (xx.x) | xx (xx.x) |
|  | Grade 2 | xx (xx.x) | xx (xx.x) |
|  | Grade 3 | xx (xx.x) | xx (xx.x) |
|  | Grade 4 | xx (xx.x) | xx (xx.x) |
|  |  |  |  |
| Adverse Event 2 | Grade 1 | xx (xx.x) | xx (xx.x) |
|  | Grade 2 | xx (xx.x) | xx (xx.x) |
|  | Grade 3 | xx (xx.x) | xx (xx.x) |
|  | Grade 4 | xx (xx.x) | xx (xx.x) |
| Denominator of the percentage is the total number of patients in the treatment group. | | | |

**Table S8:** Summary of Protocol Violations.

| **Characteristics** | **Intervention Group (N=XXX)** | **Placebo Group (N=XXX)** |
| --- | --- | --- |
|  |  |  |
| Protocol Violations | xx (xx.x) | xx (xx.x) |
| xxxxxxx | xx (xx.x) | xx (xx.x) |
| xxxxxxx | xx (xx.x) | xx (xx.x) |
|  |  |  |
| Reasons for protocol violations |  |  |
| xxxxxxx | xx (xx.x) | xx (xx.x) |
| xxxxxxx | xx (xx.x) | xx (xx.x) |
|  |  |  |
| Any consequences |  |  |
| Yes | xx (xx.x) | xx (xx.x) |
| No | xx (xx.x) | xx (xx.x) |
| Denominator of the percentage is the total number of patients in the treatment group. | | |
