# Supplementary material for: Treatment of Middle East respiratory syndrome with a combination of lopinavir/ritonavir and interferon-β1b (MIRACLE trial): statistical analysis plan for a recursive two-stage group sequential randomized controlled trial
Source: Trials. 2020 Jan 3;21:8. doi: 10.1186/s13063-019-3846-x (PMC6942374; doi:10.1186/s13063-019-3846-x)
Supplement: Supplementary file 2 — Additional file 2. Statistical analysis plan document. [file 13063_2019_3846_MOESM2_ESM.docx]

**Supplement: Statistical Analysis Plan Document for the MIRACLE trial (Treatment of Middle East Respiratory Syndrome with a combination of lopinavir/ritonavir and interferon-β1b)**

**Table of Contents**

**LIST OF ABBREVIATIONS**

[1. INTRODUCTION 4](#_Toc12447165)

[2. STUDY Outcomes 4](#_Toc12447166)

[2.1. Primary Outcome 4](#_Toc12447167)

[2.2. Secondary Outcomes 4](#_Toc12447168)

[3. STUDY DESCRIPTION 7](#_Toc12447169)

[3.1. Study Design 7](#_Toc12447170)

[3.2. Randomization Procedure 7](#_Toc12447171)

[3.3. Sample Size 7](#_Toc12447172)

[3.4. Study Interventions 8](#_Toc12447173)

[3.5. Study Duration 8](#_Toc12447174)

[3.6. Schedule of Assessments 9](#_Toc12447175)

[4. INCLUSION, EXCLUSION AND WITHDRAWAL CRITERIA 9](#_Toc12447176)

[4.1. Inclusion Criteria 9](#_Toc12447177)

[4.2. Exclusion Criteria 10](#_Toc12447178)

[5. STUDY END POINTS AND DEFINITIONS 10](#_Toc12447179)

[6. STUDY POPULATIONS 15](#_Toc12447180)

[7. STATISTICAL CONSIDERATIONS 15](#_Toc12447181)

[7.1. Statistical Significance 15](#_Toc12447182)

[7.2. Handling Dropouts and Missing Data 16](#_Toc12447183)

[7.3. Adjustment for multiplicity 16](#_Toc12447184)

[7.4. Statistical Software 16](#_Toc12447185)

[8. DESCRIPTION OF TABLES AND FIGURES 16](#_Toc12447186)

[8.1. Subject Disposition 16](#_Toc12447187)

[8.1.1. Subject Disposition 16](#_Toc12447188)

[8.1.2. Protocol Violations/ Deviations 17](#_Toc12447189)

[8.2. Baseline and Demographic Characteristics 17](#_Toc12447190)

[8.3. Efficacy Analysis 17](#_Toc12447191)

[8.3.1. Analysis of the primary outcome and continuous planning of the trial 17](#_Toc12447192)

[8.3.2. Secondary analyses of the primary outcome, secondary outcomes and subgroups 19](#_Toc12447193)

[8.4. Safety Analysis 20](#_Toc12447194)

[8.4.1. Adverse Events (AE) 20](#_Toc12447195)

[8.4.2. Vital Signs 20](#_Toc12447196)

[8.4.3. Laboratory Parameters 20](#_Toc12447197)

[8.4.4. Treatment Exposure and Compliance 21](#_Toc12447198)

[8.4.5. Prior and Concomitant Medications 21](#_Toc12447199)

[9. PHARMACOKINETIC ANALYSIS 21](#_Toc12447200)

[10. INTERIM REPORT DETAILS 21](#_Toc12447201)

[11. DATA MONITORING COMMITTEE CHARTER 22](#_Toc12447202)

[12. REPORT GENERATION 23](#_Toc12447203)

[13. CLINICAL STUDY REPORT APPENDICES 24](#_Toc12447204)

[14. REFERENCES 48](#_Toc12447205)

**LIST OF ABBREVIATIONS**

**APACHE II:**Acute Physiology and Chronic Health Evaluation II

**CONSORT**: Consolidated Standards of Reporting Trials

**CTCAE**: Common Terminology Criteria for Adverse Events

**DSMB**: Data and Safety Management Board

**ECMO:** Extra Corporeal Membrane Oxygenation

**MERS:**Middle East Respiratory Syndrome

**MERS-CoV:**Middle East Respiratory Syndrome coronavirus

**MIRACLE trial:**the *M*ERS-CoV *I*nfection t*R*eated with *A C*ombination of *L*opinavir/ritonavir and int*E*rferon β1b

**NIH:** National Institutes of Health

**SFDA:** Saudi Food and Drug Authority

**SAP:**Statistical Analysis Plan

**SOFA**: Sequential-Organ-Failure Assessment

**TEAE:** Treatment Emergent Adverse Events

# INTRODUCTION

The purpose of the Statistical Analysis Plan (SAP) is to provide a framework that addresses the protocol objectives in a statistically rigorous fashion, with minimized bias or analytical deficiencies.  Specifically, this plan has the following purpose: To prospectively (a priori) outline the types of analyses and data presentations that will addresses the study objectives outlined in the protocol, and to explain in detail how the data will be handled and analyzed, adhering to commonly accepted standards and practices.

The final study report will follow the guidelines of the Consolidated Standards of Reporting Trials (CONSORT) for reporting randomized controlled trials. [1, 2]

The trial is being conducted according to the standard requirements of Good Clinical Practice E6.[3] The SAP is developed in accordance with the International Council for Harmonisation guidelines (E9 Statistical principles for clinical trials and E3 clinical study reports guidelines)[4, 5] and with Guidelines for the Content of Statistical Analysis Plans in Clinical Trials.[6]

# STUDY Outcomes

# Primary Outcome

1. 90-day mortality

# Secondary Outcomes

**Clinical**

1. Mortality in the ICU, mortality in the hospital and 28-day mortality

2. Sequential Organ Failure Assessment (SOFA) scores at baseline and on study days 1, 3, 7, 14, 21 and 28.

3. Organ support, according to the number of days within the first 28 days after enrollment when patients do not receive specific forms of support:

- - 1. Supplemental oxygen-free days
    2. Renal replacement therapy-free days
    3. Vasopressor-free days
    4. Invasive or non-invasive mechanical ventilation free days
    5. Organ support-free days (that is, days free of invasive mechanical ventilation, renal replacement therapy and vasopressors)
    6. Extracorporeal circulation support-free days

4. ICU-free days and hospital length of stay:

1. ICU-free days are the number of days when patients are not being cared for in the ICU during the first 28 days after enrollment. Patients who die within 28 days will be assigned the value “0”
2. The hospital length of stay will be calculated as the number of days between admission and discharge from the hospital. Because of the competing risk effect of death on length of stay, length of stay will be also reported for survivors alone.

**Laboratory**

1. Viral replication kinetics will be calculated by determining viral RNA loads as reflected by Ct values by semi-quantitative PCR in serial samples of upper and lower respiratory tract secretions and blood, collected twice per week. Quantitative PCR, which may be more sensitive than Ct values, will be used if it becomes available during the trial

2. Time to clearance from the lower respiratory tract will be determined from the results of RT-PCR analyses of lower respiratory tract secretions or nasopharyngeal swabs in non-intubated patients who are unable to give sputum samples; clearance will be defined as two negative RT-PCR results not followed by a positive one. Patients who die before clearance will be censored at the time of death.

3. Planned sub-studies will examine cytokine, chemokine and immune responses in serially collected blood samples. A detailed protocol of this sub-study will be published separately.

**Safety**

1. Safety outcomes will be assessed from serious adverse event reports. The following serious adverse events will be documented at any time during the study period:

(a) acute pancreatitis (defined as having two of the following three features: (1) abdominal pain consistent with acute pancreatitis; (2) serum lipase or amylase at least three times greater than the upper limit of normal; and (3) characteristic findings of acute pancreatitis on contrast enhanced computed tomography) or magnetic resonance imaging

(b) elevation of ALT to more than five-fold upper normal limit

(c) anaphylaxis

(d) bleeding diathesis (INR > 3 without anticoagulant therapy)

2. The following data regarding adverse drug reactions will be assessed with the treating team and recorded daily for 14 days and then at 21 days and 28 days after enrollment:

(a) Allergic or sensitivity/hypersensitivity reactions, including rash, urticaria, tongue edema, bronchospasm, dyspnea and skin necrosis at the injection site

(b) Gastrointestinal signs or symptoms, including nausea, vomiting, abdominal pain and diarrhea

(c) Neurological symptoms, including fatigue, headache, insomnia, psychosis, depression and mania.

**Functional**

Data will be collected at baseline before the current illness and at 90 days and may require telephone interviews with the patients or next of kin to determine the patients’ status according to the Karnofsky Performance Scale, which is a scale from 100 (indicating “Normal,” no complaints; no evidence of disease) to 0 (indicating death).

# STUDY DESCRIPTION

# Study Design

MIRACLE trial is a recursive, two-stage, group sequential, multicenter, randomized, placebo-controlled, double-blind trial.

# Randomization Procedure

Patients will be randomly assigned to one of the two intervention arms by a stratified block randomization, which will ensure that the two arms contain equal numbers of patients. Randomization will be stratified according to center and according to whether the patients require mechanical ventilation (invasive or non-invasive) at the time of enrollment.

# Sample Size

The trial is designed as recursive, two-stage, group sequential randomized trial. The trial is designed initially to have 2 two-stage components with two interim analyses and one final analysis. The first interim analysis will be conducted when 34 subjects (17 per group) have completed 90 days of follow-up, which is about 17.5% of the total sample size needed for the classical design (a classic two-group design requires a total of 194 subjects (97 subjects per group) to have a 80% power at a significant level of 2.5% (one sided test) to detect 20% absolute risk reduction in 90 days mortality among subjects receiving treatment (20%) compared to control group (40%)). Re-estimation of sample size based on the observed effect size will be calculated by using the following formula assuming a conditional power of 80% to decide if the trial should continue:

$$n12={[\frac{\sqrt{2}\sigma}{\delta}(\theta^{-1}\left( 1-\alpha12+p\text{11} \right)-\theta^{-1}\left( 1-Pc \right))]}^{2}$$

Based on the estimated sample size we will recalculate the conditional error and set the parameters for the second two-sage. At the second interim analysis, should the trial continue for efficacy sample size readjustment will be made based on the previous formula and new boundaries will be calculated for the final stage analysis.

# Study Interventions

The intervention group will receive the standard of care as well as lopinavir/ritonavir and recombinant IFN-β1b. Lopinavir/ritonavir (400 lopinavir mg/100 mg ritonavir) will be administered every 12 h for 14 days in tablet form. For patients who are unable to take medications by mouth, the lopinavir/ritonavir (400 lopinavir mg/100 mg ritonavir) will be administered as a 5-ml suspension every 12 h for 14 days via a pre-existing or newly placed nasogastric tube. IFN-β1b will be administered as 0.25-mg/ml subcutaneous injections on alternate days for 14 days (for a total of seven doses).

The control group will receive standard of care as well as placebo treatment for 14 days at the same frequency as the intervention group, to maintain blinding. One placebo will be given every 12 h and will comprise a sucrose tablet or capsule, or 5 ml of normal saline via nasogastric tube for patients who are unable to take medications by mouth. Patients in the control group will receive 1 ml of normal saline by subcutaneous injection on alternate days.

# Study Duration

The study interventions will continue for 14 days or until hospital discharge. Patients will be followed up daily until day 28 or hospital discharge and then at day 90.

# Schedule of Assessments

# INCLUSION, EXCLUSION AND WITHDRAWAL CRITERIA

# Inclusion Criteria

The trial includes hospitalized MERS patients who are 18 years old or older with laboratory confirmation of MERS-CoV infection, in addition to evidence of acute organ dysfunction that is judged related to MERS.

1. Adult (defined as ≥ 18 years of age)

2. Laboratory confirmation of MERS-CoV infection by reverse-transcription polymerase chain reaction (RT-PCR) from any diagnostic sampling source, and

3. New organ dysfunction that is judged to be related to MERS including: hypoxia defined as requirement of supplemental oxygen to maintain oxygen saturations > 90%, hypotension (systolic blood pressure < 90 mmHg) or the need for vasopressor/inotropic medication, renal impairment (increase of creatinine by 50% from baseline, glomerular filtration rate reduction by > 25% from baseline or urine output of < 0.5 ml/kg for 6 h – risk stage by RIFLE criteria), neurological pathology (reduction of Glasgow Coma Scale by 2 or more, i.e., 13 or lower of 15 points), thrombocytopenia (<150,000 platelets/mm3) or gastrointestinal symptoms that require hospitalization (e.g., severe nausea, vomiting, diarrhea or/and abdominal pain).

# Exclusion Criteria

Patients are excluded from participation in the study if they meet any of the following exclusion criteria:

1. Suicidal ideation based on history (contraindication to IFN-β1b) 2. Known allergy or hypersensitivity reaction to lopinavir/ritonavir or to recombinant IFN-β1b, including, but not limited to, toxic epidermal necrolysis, Stevens-Johnson syndrome, erythema multiforme, urticaria or angioedema 3. Elevated alanine aminotransferase (ALT) more than five-fold the upper limit in the hospital’s laboratory

2. Use of medications that are contraindicated with lopinavir/ritonavir and that cannot be replaced or stopped during the study period, such as CYP3A inhibitors

3. Pregnancy – eligible and consenting female participants of childbearing age will be tested for pregnancy before enrollment in the study 6. Known HIV infection, because of concerns about the development of resistance to lopinavir/ritonavir if used without combination with other anti-HIV drugs, or

4. Patient likely to be transferred to a non-participating hospital within 72 h.

# STUDY SECONDARY END POINTS AND DEFINITIONS

| **Outcome** | **Definition** |
| --- | --- |
| 28-day mortality | Death from any cause within 28 days of enrollment |
| Hospital mortality | Death from any cause in the index hospitalization |
| ICU mortality | Death from any cause in index ICU admission. |
| Sequential Organ Failure Assessment scores | SOFA score on study days 1, 3, 7, 14, 21 and 28 |
| Supplemental oxygen-free days | Number of days within the first 28 days after enrollment when patients do not receive of supplemental oxygen. Patients who die within 28 days will be assigned the value “0” |
| Renal replacement therapy-free days | Number of days within the first 28 days after enrollment when patients do not receive ofrenal replacement therapy. Patients who die within 28 days will be assigned the value “0” |
| Vasopressor-free days | Number of days within the first 28 days after enrollment when patients do not receive ofvasopressors. Patients who die within 28 days will be assigned the value “0” |
| Invasive or non-invasive mechanical ventilation-free days | Number of days within the first 28 days after enrollment when patients do not receive ofmechanical ventilation. Patients who die within 28 days will be assigned the value “0” |
| Organ support-free days | Number of days within the first 28 days after enrollment when patients do not receive ofinvasive mechanical ventilation, renal replacement therapy and vasopressor. Patients who die within 28 days will be assigned the value “0” |
| Extracorporeal circulation support-free days | Number of days within the first 28 days in which patients are not receiving extracorporeal circulation support. Patients who die within 28 days will be assigned the value “0” |
| ICU-free days | Number of days in which patients are not being cared for in the ICU during the first 28 days after enrollment. Patients who die within 28 days will be assigned the value “0” |
| Post-randomization hospital length of stay | Number of days between randomization and discharge from the hospital. Because of the competing risk effect of death on length of stay, length of stay will be also reported for survivors alone |
| Renal replacement therapy at day 90 | Number and percentage of patients on renal replacement therapy at day 90 |
| Oxygen supply at day 90 | Number and percentage of patients on oxygen supply at day 90 |
| Non-invasive mechanical ventilation at day 90 | Number and percentage of patients on non-invasive mechanical ventilation at day 90 |
| Invasive mechanical ventilation at day 90 | Number and percentage of patients on Invasive mechanical ventilation at day 90 |
| Secondary laboratory outcomes | |
| Viral replication kinetics | upE and ORF1cycle thresholds of blood and respiratory samples |
| Time to clearance from the lower respiratory tract | Number of days from randomization to MERS-CoV RNA clearance of respiratory samples defined as two negative RT-PCR results not followed by a positive one. Patients who die before clearance will be censored at the time of death |
| Safety outcomes | |
| Serious adverse event reports (SAE) | The number and percentage of reported serious adverse events any time during the study period. These SAEs include: acute pancreatitis, severe elevation of Alanine aminotransferase(ALT) to more than five-fold the upper normal limit, anaphylaxis, bleeding diathesis and others |
| Adverse Events | The number and percentage of adverse events graded using the Common Terminology Criteria for Adverse Events, at any time within 28 days after enrollment. The adverse drug reactions include: allergic reactions, gastrointestinal, general nervous system and others. |
| Functional outcomes | |
| Karnofsky score | Karnofsky Performance Status Scale for functional impairment, which is a scale from 100 (indicating “Normal,” no complaints; no evidence of disease) to 0 (indicating death) |

# STUDY POPULATIONS

**Intention-to-treat population**

The Intention-to-treat population consists of all enrolled patients whether or not they received the allocated intervention, and will be used for the primary analysis.

**Per-protocol population**

Per protocol analysis is defined as the patients who received the allocated interventions (defined by any dose of the study intervention) and without major protocol violations.

| **ENDPOINTS/ ANALYSIS** | **POPULATION /ANALYSIS SETS** |
| --- | --- |
| Demographic and Baseline Characteristics | Intention-to-treat population |
| Efficacy Analysis | Intention-to-treat population and/or Per-protocol Population |
| Safety Analysis | Intention-to-treat population |

# STATISTICAL CONSIDERATIONS

# Statistical Significance

Hypothesis testing for primary outcome will be done using one-sided, 0.05 level of significance.

Statistical tests for variables other than the primary outcome will be performed using a two -sided alpha value of 5% to denote significance level.

As appropriate, the Chi-square test or Fisher’s exact test will be used to compare the categorical variables, which will be reported as numbers and percentages. Student’s t-test or the Mann–Whitney U Test will be used as appropriate to compare the continuous variables, which will be reported as means and standard deviations or as medians and interquartile ranges.

# Handling Dropouts and Missing Data

All missing data will be reviewed and characterized in terms of their pattern (eg. Missing completely at random, missing at random, etc.). For missing completely at random, all analyses will be based on list-wise deletion approach where observation will complete values will be only considered for analysis. For variables with values missing at random, multiple imputation techniques will be utilized to impute the missing values as suggested by Rubin’s (1987).

# Adjustment for multiplicity

To adjust for multiple testing, we will use the False Discovery Rate (FDR) as described by Benjamini and Hochberg [13]. In this procedure all hypothesis tests will be sorted in an ascending order based on their calculated p-value. All hypothesis tests below an index K will be rejected where K calculated as follows:

$$K=max\left\{ i:p\left( i \right)\leq\frac{i}{m}.q \right\}$$

*Where* i=m, …1 ; m is the number total number of tested hypotheses ; q =.05.

# Statistical Software

All analyses including the interim and final analysis will be analyzed using SAS version 9.4 NC.

# DESCRIPTION OF TABLES AND FIGURES

# Subject Disposition

# Subject Disposition

The number and percentage of randomized patients to each group will be reported. We will report the number of randomized patients who received the interventions. We will also report the number of screened patients (defined as all hospitalized patients with MERS), who met the eligibility criteria but not enrolled and reasons for non-enrollment. A CONSORT flow diagram of the trial progress will be constructed.

# Protocol Violations/ Deviations

Potential violations that may result in the exclusion of a patient from the Per-Protocol population include:

- Patient compliance with study medication is <80%,
- Premature discontinuation of treatment

# Baseline and Demographic Characteristics

All analyses will be performed using SAS 9.4 with specially written code for the analysis of the primary outcome that accounts for the recursive design as described in [Chang, et al.][3]. We will summarize and report the demographics and baseline clinical characteristics using descriptive statistics. Baseline characteristics will be presented in for the two study groups including age, sex and body mass index, the presence of co-infections, nosocomial versus community -acquired MERS infection, acute physiology and chronic health evaluation (APACHE) II scores[11], Sequential Organ Failure Assessment scores and the KarnofskyPerformance Status Score. We will report co-morbidities and the interventions received before randomization for patients in each group. We will report baseline laboratory values (international normalization ratio ,platelet count, hemoglobin, white blood cell count, lymphocyte count, liver enzymes, glucose, serum amylase, blood urea nitrogen , creatinine, creatine kinase , lactate, serum cortisol, adrenocorticotropic hormone and thyroid function tests), respiratory and vital parameters in addition to the location of the patient at time of randomization. Categorical variables will be summarized by treatment group using number (n) and percentage (%). Descriptive statistics such as mean, standard deviation (SD) or median (Q1, Q3) will be calculated for continuous variables.

# Efficacy Analysis

# Analysis of the primary outcome and continuous planning of the trial

The primary outcome of the current trial is 90-day mortality. The primary outcome is defined as all-cause mortality after enrollment in the trial within 90-day, as either an inpatient or outpatient and which will be calculated as the proportion of people who have died within 90 days of treatment administration and will be analyzed at each stage using the Z test for difference in proportion using the following statistic:

$Z=\frac{\delta}{\sigma}\sqrt{\frac{n_{ki}}{2}}$, where

$\sigma=\sqrt{\left[ r_{1}\left( 1-r_{1} \right)+r_{2}\left( 1-r_{2} \right) \right]/2}$,

$$\delta=r_{1-}r_{2}$$

Where $r_{1}$ , $r_{2}$ are the proportion of standard of care and treatment group respectively, $n_{ki}$ is the sample size per group for k^th^ stage of the two-stage design, and i=1,2 is the stage index of the two stages.

In the interim analysis (i.e. at each i=1 of the k two stages) ,the primary outcome will be evaluated and the trial sample size will be re-estimated for the subsequent stage based on the observed effect size using the following formula assuming a conditional power of 80% ($Pc=0.8)$ to decide if the trial should continue:

$$n_{k,2}={[\frac{\sqrt{2}\sigma}{\delta}(\theta^{-1}\left( 1-\alpha_{1,2}+p_{1,1} \right)-\theta^{-1}\left( 1-Pc \right))]}^{2}$$

At the first interim analysis shall the data suggest that another stage of two-stages is required, we will recalculate the conditional error and new boundaries will be calculated for K=2 as follows:

$$A\left( p_{k,1} \right)=\alpha_{k+1,1}+\alpha_{k+1,2}\left( \beta_{k+1,1}- \alpha_{k+1,1} \right)-\frac{1}{2}\left( \beta_{k+1,1}^{2}-\alpha_{k+1,1}^{2} \right), k=0,1,\ldots K$$

Where $A\left( p_{0,1} \right)$ is type I error which is set to 0.05 the new $\alpha_{k+1,2}$boundary for the k^th^+1 stage for pre chosen $\beta_{k+1,1}, \alpha_{k+1,1}$ will be calculated as follows:

$$\alpha_{k+1,2}=\frac{A\left( p_{k,1} \right)+\frac{1}{2}\left( \beta_{k+1,1}^{2}-\alpha_{k+1,1}^{2} \right)-\alpha_{k+1,1}}{\beta_{k+1,1}- \alpha_{k+1,1}}$$

At the end of the trial, the treatment will be declared efficacious if the calculated stage-wise ordered p-value $p_{k,2}$ is less than $\alpha_{k,2}$. The adjusted p-value will be obtained using the following equations:

$$\left\{ \begin{aligned} P_{K_{0}-1,2}=\left\{ \begin{aligned} t for k=1, \\ \alpha_{k_{0},1}+t\left( \beta_{k_{0},1}- \alpha_{k_{0},1} \right)-\frac{1}{2}\left( {\beta^{2}}_{k_{0},1}-{\alpha^{2}}_{k_{0},1} \right) for k=2, \end{aligned} \right. \\ P_{i-1,2}= \alpha_{i,1}+\left( p_{i,1}+ p_{i,2} \right)\left( \beta_{i,1}- \alpha_{i,1} \right)-\frac{1}{2}\left( {\beta^{2}}_{i,1}-{\alpha^{2}}_{i,1} \right) for i=1, \ldots{,K}_{0-1} \end{aligned} \right.$$

Where K_0_ is the total number of two-stage stages, and *t* is the sum of stage wise raw p-values. Finally the adjusted 95% one sided confidence interval will be calculated by solving the following equation numerically:

$$N\left( \frac{\delta_{k_{0},2}}{\sigma}\sqrt{\frac{n_{k0,1}}{2}}-z_{1-p_{k0,1}} \right)+ N\left( \frac{\delta_{k_{0},2}}{\sigma}\sqrt{\frac{n_{k0,2}}{2}}-z_{1-p_{k0,2}} \right)=\alpha_{k_{0},2}$$

Then the 95% one sided confidence bound will be given by

$$\delta_{c}=\max_{1\leq i\leq k_{0-1}} \left\{ \delta_{i,1}, \delta_{k_{0},k} \right\}$$

# Secondary analyses of the primary outcome, secondary outcomes and subgroups. A secondary adjusted analysis will be conducted using multiple logistic regression analysis in which death within 90 days will be modeled as the dependent variable and a set of baseline variables that are strongly believed to affect the outcome of MERS-CoV infection will include as independent variables. These independent variables will include at minimum age, community-acquired versus hospital-acquired infection, mechanical ventilation, center, and Sequential-Organ-Failure Assessment (SOFA) score[12]. Ninety-day median survival time will be summarized and reported using Kaplan–Meier survival curves and will be compared between the study groups using the log-rank test.

Analysis of secondary outcomes including safety outcomes will be compared in the intention-to-treat cohort and will be reported as relative risk with the corresponding 95% CI.

Subgroup analyses will be conducted if patient numbers permit (e.g., no fewer than five patients in subgroups of interest) in a priori defined subgroups (Mechanical ventilation vs No mechanical ventilation at day 0, High APACHE II vs Low APACHE II at day 0, Vasopressors vs No vasopressors at day 0, Renal replacement therapy vs No renal replacement therapy at day 0, ≤ 7 days between onset of symptoms to enrollment vs > 7 days between onset of symptoms to enrollment). Multivariable logistic regression will be used to report the results of tests of interactions for these subgroups.

SOFA scores at each visit will be summarized for each group and will be compared using Mann–Whitney U Test.

Karnofsky score at Day 90 will be summarized for each group and will be compared using Mann–Whitney U Test.

# Safety Analysis

# Adverse Events (AE)

All adverse events will be grouped using Common Terminology Criteria for Adverse Events (CTCAE) V4 of National Institutes of Health (NIH). Adverse events will be grouped into 5 aggregate groups and reported for the entire study period. All results will be summarized in terms of frequency and percentage and will be compared across study arms using Fisher exact test. All results will be declared statistically significant with P-value <0.05.

Serious adverse events (Acute pancreatitis, severe elevation of ALT to more than five-fold the upper normal limit , Anaphylaxis , Bleeding diathesis, Others) will be summarized by treatment group using number (n) and percentage (%). Relationship with study drugs will also be summarized. Serious adverse events related to the study drugs will be summarized by treatment group using number (n) and percentage (%).

The classification of Adverse Events in the MIRACLE Trial (MERS-CoV Infection Treated with a Combination of Lopinavir / Ritonavir and Interferon Beta 1B) using the NIH Common Terminology Criteria for Adverse Events (CTCAE), Version 4.0 described in Table S6.

# Vital Signs

Vital signs such as temperature (^◦^C), respiratory rate, systolic blood pressure (mmHg), highest heart rate, lowest mean arterial pressure (mmHg) will be summarized using median (Q1, Q3) at baseline and over time.

# Laboratory Parameters

Laboratory values such as international normalization ratio, platelet count, hemoglobin, white blood cell count, lymphocyte count, liver enzymes, glucose, serum amylase, blood urea nitrogen, creatinine, creatine kinase, lactate, serum cortisol, adrenocorticotropic hormone, thyroid function tests Glasgow coma scale score and Urine output (mL/d) will be summarized using median (Q1, Q3) at baseline. Figures will be presented for the serial measurements Day 1, Day 3, Day 7, Day 14, Day 21 and Day 28.

# Treatment Exposure and Compliance

For each group we will report the time of hospital admission to randomization and the time of randomization to the first dose received of the study drugs. We will report the received and duration of study intervention for each group, in addition to the missing or incomplete doses and protocol violations.

# Prior and Concomitant Medications and Interventions

We will summarize any use of vasopressors, renal replacement therapy, neuromuscular blockade, mechanical ventilation, extra corporeal membrane oxygenation (ECMO), nitric oxide, prone ventilation and tracheostomy. Also, we will summarize the use of Intravenous immunoglobin, antiviral therapy, antibiotics, corticosteroids and statins.

Dose of vasopressors (Dopamine (ug/kg/min), norepinephrine (ug/kg/min), epinephrine (ug/kg/min), phenylephrine (ug/kg/min), vasopressin (U/min), dobutamine (ug/kg/min), milrinone (ug/kg/min)) and duration of corticosteroid (days) use will be summarized by treatment group using median (Q1, Q3).

# PHARMACOKINETIC ANALYSIS

Pharmacokinetic analysis will not be done for this study.

# INTERIM REPORT DETAILS

**DSMB & Interim analyses**

A detailed interim analysis plan is reported in the MIRACLE protocol. The trial is designed as recursive, two-stage, group sequential randomized trial. The trial is designed initially to have 2 two-stage components with two interim analyses and one final analysis. The first interim analysis will be conducted when 34 subjects (17 per group) have completed 90 days of follow-up, which is about 17.5% of the total sample size needed for the classical design (a classic two-group design requires a total of 194 subjects (97 subjects per group) to have a 80% power at a significant level of 2.5% (one sided test) to detect 20% absolute risk reduction in 90 days mortality among subjects receiving treatment (20%) compared to control group (40%)). A Data and Safety Management Board (DSMB) will be convened to review the unblinded data (efficacy and safety) and advice on continuation or termination of the trial. The determination of the stopping boundaries in the first two-stage design was calculated using the conditional power method based on summing stage-wise p values. At the first interim analysis the DSMB will determine whether the trial should be terminated for futility or not using the following boundaries and their corresponding decisions

**Continuous planning of the trial:** Re-estimation of sample size based on the observed effect size will be calculated by using the following formula assuming a conditional power of 80% to decide if the trial should continue:

$$n12={[\frac{\sqrt{2}\sigma}{\delta}(\theta^{-1}\left( 1-\alpha12+p\text{11} \right)-\theta^{-1}\left( 1-Pc \right))]}^{2}$$

Based on the estimated sample size we will recalculate the conditional error and set the parameters for the second two-sage. At the second interim analysis, should the trial continue for efficacy sample size readjustment will be made based on the previous formula and new boundaries will be calculated for the final stage analysis.

Stopping boundaries in the MIRACLE trial

| **Boundary** | **Value** | **Decision** |
| --- | --- | --- |
| Efficacy sopping boundary (α_1_) | 0 | No stopping for efficacy |
| Futility stopping boundary (β_1_) | 0.2 | Stop the trial for futility if less than stage-wise P-value |
| Efficacy stopping boundary (α_2_) | 0.2250 | Stop trial for efficacy at the second stage or recalculate based on conditional power at first interim analysis |

# DATA MONITORING COMMITTEE CHARTER

The Data Safety Monitoring Board, which is responsible for reviewing reports regarding the safety of the study patients and protocol adherence, may make recommendations to continue or terminate the study on the basis of the results from the interim analysis. The board will meet at the beginning of the study and at 6-monthly intervals thereafter, or as needed.

# REPORT GENERATION

**Figure Legend**

**Figure 1:** CONSORT flow chart for the MIRACLE trial

**Supplemental Table Legends**

**Supplemental Table 1.** Baseline characteristics – ITT population.

**Supplemental Table 2.** Summary of interventions and co-interventions.

**Supplemental Table 3.** Primary Outcome: 90-day mortality.

**Supplemental Table 4.** Secondary Outcomes.

**Supplemental Table 5.** Subgroup analyses.

**Supplementary Table 6.** Classification of Adverse Events in the MIRACLE Trial (MERS-CoV Infection Treated with a Combination of Lopinavir / Ritonavir and Interferon Beta 1B) using the NIH Common Terminology Criteria for Adverse Events (CTCAE), Version 4.0.

**Supplemental Table 7:** Summary of Adverse Events by severity.

**Supplemental Table 8:** Summary of Protocol Violations.

**Supplemental Figure 2:** Kaplan Meier Survival Curve for overall survival

**Supplemental Figure 3:** Physiologic parameters among patients treated with the Treatment group and Control group.

# CLINICAL STUDY REPORT APPENDICES

# REFERENCES

1. Moher D, Hopewell S, Schulz KF, Montori V, Gotzsche PC, Devereaux PJ, Elbourne D, Egger M, Altman DG: CONSORT 2010 explanation and elaboration: updated guidelines for reporting parallel group randomised trials. *BMJ* 2010, 340:c869.

2. Schulz KF, Altman DG, Moher D, Group C: CONSORT 2010 statement: updated guidelines for reporting parallel group randomised trials. *BMJ* 2010, 340:c332.

3. International Conference on Harmonisation of Technical Requirements for Registration of Pharmaceuticals for Human Use: Good Clinical Practice (GCP) Guideline [<http://www.ich.org/fileadmin/Public_Web_Site/ICH_Products/Guidelines/Efficacy/E6/E6_R2__Step_4_2016_1109.pdf>]

4. The International Council for Harmonisation of Technical Requirements for Pharmaceuticals for Human Use (ICH): STATISTICAL PRINCIPLES FOR CLINICAL TRIALS [<http://www.ich.org/fileadmin/Public_Web_Site/ICH_Products/Guidelines/Efficacy/E9/Step4/E9_Guideline.pdf>]

5. International Conference on Harmonisation of Technical Requirements for Registration of Pharmaceuticals for Human Use: E3 - Structure and content of clinical study reports [<http://www.ich.org/fileadmin/Public_Web_Site/ICH_Products/Guidelines/Efficacy/E3/E3_Guideline.pdf>]

6. Lauvrak V, Hafstad E, Fure B: In *Intermittent Pneumatic Compression to Prevent Venous Thromboembolism in Hospitalized Patients: Systematic Scoping Review.* Oslo, Norway; 2016: *NIPH Systematic Reviews: Executive Summaries*].

# 
